# Change of Ownership and Quality of Home Health Agency Care

**DOI:** 10.1001/jamahealthforum.2024.3767

**Published:** 2024-11-01

**Authors:** Zhanji Zhang, Kun Li, Siyi Wang, Shekinah Fashaw-Walters, Yucheng Hou

**Affiliations:** 1 Department of Applied Economics, University of Minnesota, Minneapolis; 2 Duke-Margolis Institute for Health Policy, Duke University, Washington, DC; 3 Department of Economics, Rice University, Houston, Texas; 4 Department of Medical Ethics and Health Policy, Perelman School of Medicine, University of Pennsylvania, Philadelphia; 5 Department of Management, Policy and Community Health, School of Public Health, The University of Texas Health Science Center at Houston

## Abstract

**Importance:**

The home health agency (HHA) market has seen increasingly more change in ownership transactions. Little is known about the organizational characteristics and quality of care of HHAs after ownership has changed.

**Objective:**

To examine whether an HHA change in ownership was associated with different quality-of-care outcomes, patient volume, and staffing levels.

**Design, Setting, and Participants:**

Using current HHA change of ownership files linked to publicly available Medicare HHA data, this staggered difference-in-differences analysis evaluated ownership change transactions of Medicare-certified HHAs from quarter 1 of 2016 to quarter 4 of 2019. Pretransaction and posttransaction HHA characteristics and quality-of-care outcomes were compared between HHAs that changed ownership and up to 8 matched controls. The transactions in the main analysis included (1) when an HHA remained as for-profit with a change in ownership and (2) when an HHA changed from nonprofit/public to for-profit ownership. The data were analyzed between November 2023 and September 2024.

**Main Outcomes and Measures:**

The primary outcomes were HHA-quarter measures of star ratings, the individual quality measures that compose the star ratings based on the Outcome and Assessment Information Set, and claims-based quality metrics, such as hospitalizations and emergency department visits. The secondary outcomes included HHA year measures of Medicare patient volume, per capita payments, and staffing levels (full-time equivalents and minutes per visit).

**Results:**

The main dataset included 294 Medicare-certified HHAs that changed ownership, matched with 2330 controls. In 3 years after an ownership change, quarterly star ratings increased by 0.18 (95% CI, 0.05-0.31) relative to matched controls, with greater increases among HHAs that converted from nonprofit/public to for-profit. No significant improvement was observed in the 60-day rates of hospital admissions or outpatient emergency department visits. Further, no significant changes were observed in the number of Medicare beneficiaries, but per capita payments increased within 2 years post–ownership change. Significant reductions were observed in full-time equivalents of registered nurses (−17% [95% CI, −31% to −3%]) and home health aides (−26% [95% CI, −39% to −13%]), as well as per-visit minutes for skilled nursing care (−5% [95% CI, −9% to −1%]), physical therapy (−3% [95% CI, −5% to 0%]), and home health aide care (−11% [95% CI, −15% to −6%]).

**Conclusions and Relevance:**

In this difference-in-differences analysis of Medicare-certified HHAs, ownership change was associated with higher star ratings and Medicare per capita payments, but not with claims-based quality measures. Reduction in staffing levels after ownership change raises concerns about implications for quality of care.

## Key Points

**Question:**

Was a change of ownership for home health agencies (HHAs) associated with different quality-of-care outcomes, patient volume, and staffing levels?

**Findings:**

This difference-in-differences analysis of 294 Medicare-certified HHAs that have changed ownership observed higher quarterly star ratings after a change of ownership, especially among HHAs converted from nonprofit/public to for-profit organizations; however, ownership change was not associated with claims-based quality metrics, such as hospitalizations and emergency department visits. Medicare per capita payments in the first 2 years after ownership change were higher, and staffing levels were lower.

**Meaning:**

Change of ownership was followed by changes in the organizational characteristics of HHAs, which could affect the quality of care.

## Teaser

This difference-in-differences analysis investigates changes in ownership of home health agencies in the US and associated changes in quality of care, patient volume, and staffing levels.

## Introduction

The home health industry has been rapidly growing due to population aging.^1,2^ National spending for home health agency (HHA) services increased from 93.8 billion in 2016 to 132.9 billion in 2022, with a 6.0% growth in the years after the COVID-19 pandemic,^3^ growing faster than facility-based nursing care.^3^ Meanwhile, the number of patients admitted to HHAs directly from the community is increasing substantially,^4^ in addition to patients admitted from acute care and other post–acute care settings. Due to the rising demand, low costs of entry, and low fixed costs for operating,^5^ potential high returns on HHA investment have attracted considerable attention from buyers. HHAs have increasingly changed ownership in the past decade, with 80 to 150 ownership change transactions annually.^6^ A recent report also shows that private equity firms were behind 5.7% of all HHAs certified by the Centers for Medicare & Medicaid Services (CMS).^7^

In 2022, 76% of HHAs were for-profit, 17% were nonprofit, and 7% were public (ie, government owned).^8^ Concerns have been raised that change of ownership in health care may cause operation disruptions.^7,9,10^ Additionally, certain ownership structures (eg, acquisition of nonprofit organizations) could shift organizations’ focus to short-term profit generating, potentially harming patient outcomes.^7,9^ The impact of ownership change on HHAs’ operation and quality remains unclear. Prior work on HHA ownership only focused on comparing care processes and quality between HHAs with and without for-profit status and chain ownership.^11,12^ However, this work did not examine the association between ownership change and these outcomes. Several studies examined ownership change in other health care sectors, such as all types of ownership change, conversions from nonprofit to for-profit status, and private equity and real estate investment trust acquisitions among skilled nursing facilities and nursing homes.^10,13,14,15,16^

Although evidence regarding the HHA industry is scarce, understanding the implications of ownership change is important for improving the quality of care received by the growing aging population. As HHAs provide care in home and community settings, they are expected to reach homebound populations that generally face barriers to accessing skilled nursing care or therapies, such as individuals with disabilities, those residing in rural and other medically underserved communities, or people with complex biopsychosocial needs.^17,18^ Given the home-based nature, HHA care delivery primarily depends on the direct contact between home health practitioners and patients, which is different from care provided at institutional facilities, in which factors like number of beds, occupancy rates, and facility environment may also influence the care quality. These unique features may motivate the buyers to use different strategies to improve HHAs’ profitability compared to other health care organizations, resulting in different quality outcomes.

Thus, using the current HHA change of ownership data from the CMS, we first provided current data on the frequency and features of change of ownership transactions between quarter 1 of 2016 and quarter 4 of 2019 among a nationwide sample of Medicare-enrolled HHAs. Second, we assessed the association between ownership change and a set of HHA-level measures by comparing differences in quality-of-care outcomes, patient volume, per capita payments, and staffing levels between HHAs that changed ownership with matched controls before and after the transaction. Finally, we tested whether the associations between ownership change and HHA outcomes vary by an HHA’s for-profit or nonprofit status before the transaction.

## Methods

### Study Design and Participants

The University of Texas Health Science Center at Houston institutional review board did not require review of this difference-in-differences analysis because all data were publicly available. Informed consent was also waived because the data did not identify individual patients. Adherence to the Strengthening the Reporting of Observational Studies in Epidemiology (STROBE) reporting guideline was ensured.^19^

We used the CMS HHA Change of Ownership file (October 2023 version) to identify HHA change of ownership transactions, defined as the Medicare identification number of an acquired HHA transferred from the seller (the previous owner) to the buyer (the new owner) (eFigure 1 in Supplement 1).^20^ The file contains information on transactions of Medicare-certified HHAs in and after 2016, including the enrollment identification numbers of the buyer and seller, the sold facility’s CMS certification number, and the effective date. We excluded 2 transactions in which hospice acted as sellers and 11 transactions that resulted in the dissolution of previous owners’ Medicare identification numbers because we could not consistently observe their posttransaction outcomes.

Next, we linked the Change of Ownership file with the Provider of Services database to obtain HHA’s ownership type, (1) for-profit or (2) nonprofit/public before and after ownership change to characterize the type of transactions. Specifically, we focused on (1) HHAs that maintained their for-profit status before and after a change of ownership and (2) HHAs that changed ownership from nonprofit/public to for-profit. For our difference-in-differences analyses, we excluded transactions where nonprofit/public HHAs changed ownership while remaining nonprofit/public because nonprofit/public HHAs typically do not have owners like for-profit HHAs do. A typical change of ownership for nonprofit HHAs reflects a change in the governing board members rather than ownership structures. We also excluded atypical transactions where for-profit HHAs were converted to nonprofit/public status. We further linked the file with Home Health Compare and the HHA Utilization and Payment Public Use File to obtain HHA-level characteristics and quality outcomes. For inclusion in the main analysis, an HHA must have received patient care star ratings and have nonmissing claims-based measures for at least 2 quarters. These eligibility criteria resulted in the HHAs included in our main analysis that changed ownership from 2016 to 2019 (eFigure 2 in Supplement 1).

We matched each HHA that changed ownership to up to 8 control HHAs, including for-profit and nonprofit/public HHAs that never had ownership change transactions during the study period. We used exact matching based on year, ownership type (for-profit or nonprofit/public), US census region (Northeast, Midwest, South, West), whether the agency had any branch, and whether the agency also participated in the Medicare program as a hospice. Nearest neighbor matching was further used to match the HHA size, measured by the total number of Medicare beneficiaries. All the variables used for matching were measured in the first observation year, which resulted in the group of matched controls in the main analysis. Additional eligibility criteria for control HHAs are provided in eTables 1 and 2 in Supplement 1. In the sensitivity analyses, we also conducted analyses using all control HHAs that met eligibility criteria without matching.

### Outcome Measures

The primary outcomes were HHA quarterly measures of composite patient care star rating scores, along with the 6 individual process and outcome quality measures from the Outcome and Assessment Information Set (OASIS) that compose the overall star ratings.^21^ We further supplemented the OASIS-based measures with 2 risk-adjusted claims-based measures: the percentage of episodes that had acute care hospitalizations (included in the calculation of composite star rating scores) and the percentage of episodes that had outpatient emergency department visits within 60 days of HHA admission (eTable 3 in Supplement 1). Home Health Compare is updated quarterly, with a lag of 6 to 9 months in reporting periods for these measures (eTable 4 in Supplement 1).^21^

The secondary outcomes included HHA year–level measures of service volume (number of Medicare beneficiaries and Medicare per capita payments) and measures of staffing levels (full-time equivalent and staffing minutes per visit), both logarithmically transformed to account for the skewed distributions. We examined full-time equivalents of 5 types of home health professionals (registered nurse, licensed practice nurse/licensed vocational nurse, physical therapist, occupational therapist, and home health aide). We also examined staffing minutes per visit by skilled nursing, physical therapy, occupational therapy, and home health aide services.

### Statistical Analysis

We first examined baseline characteristics of HHAs with ownership change and matched controls. For the main analysis, we used a staggered difference-in-differences design developed by Callaway and Sant’Anna^22^ to compare outcomes between HHAs with and without ownership changes, before and after the transactions, while accommodating heterogeneous treatment effects over time and across HHAs that changed ownership in different periods.^23^ We estimated an event study specification to examine outcome changes over time. All regressions controlled for HHA fixed effects and year-quarter fixed effects, to account for time-invariant HHA characteristics and national trends. We weighted each HHA-quarter or HHA year observation by the number of patients enrolled in Medicare in the first observation year of the study period. We did not include other time-varying covariates that may be affected by change of ownership transactions.^24^ For each regression, we empirically tested the parallel trend assumption up to 5 quarters or 4 years before ownership change. Given the evidence of pretrends, we further detrended the outcome variables using the estimated coefficients on a linear differential pretrend between HHAs with ownership change and matched controls. In sensitivity analysis, we also provided a set of analyses without detrending. Detailed empirical approaches are included in eMethods in Supplement 1.

Because change in ownership type and change in owners could have different impacts, we also examined the associations of ownership change with outcomes by transaction type. For transactions in which ownership changed but the type remained for-profit, the control group is the matched for-profit agencies that have never changed ownership during the study period. For transactions in which the acquired HHAs changed from nonprofit/public to for-profit, the control group is the matched nonprofit agencies that have never changed ownership during the study period. We computed 95% CIs based on standard errors clustered within HHAs across quarterly or yearly observations. A 2-sided significance threshold was set at *P* < .05. All statistical analyses were performed using Stata, version 18.0 (StataCorp), and the data were analyzed between November 2023 and September 2024.

## Results

**Figure 1.**
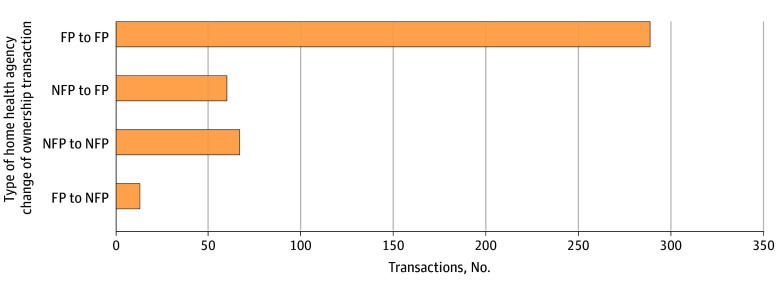
Home Health Agency (HHA) Change of Ownership Transactions by Type, 2016-2019 A total of 429 ownership change transactions were recorded in the Centers for Medicare & Medicaid Services (CMS) HHA Change of Ownership file (October 2023 version) from quarter 1 of 2016 to quarter 4 of 2019. The number of transactions by each type of home health agency change of ownership is presented in this bar graph. Ownership types were obtained by linking the CMS HHA Change of Ownership file with the Provider of Service file. We excluded 2 transactions in which hospice was the seller and 11 transactions in which ownership change resulted in the dissolution of CMS certification numbers of acquired HHAs. FP indicates for-profit; NFP, nonprofit/public.

Figure 1 shows the types of HHA ownership changes for a total of 429 transactions from 2016 to 2019 (step 3 in eTables 1 and 2 in Supplement 1). Most HHAs (n = 289) did not change their for-profit status before and after ownership change; 60 HHAs converted from nonprofit/public to for-profit status. For the main difference-in-differences analyses, 67 transactions were excluded when nonprofit/public HHAs changed ownership but remained nonprofit/public. In addition, 13 atypical transactions in which for-profit HHAs were converted to nonprofit/public status were excluded.

Table 1 shows the baseline characteristics of HHAs that changed ownership and matched controls. Before the change of ownership transactions, HHAs that changed ownership (vs matched controls) had higher star ratings (mean [SD], 3.35 [0.79] vs 3.24 [0.90]), higher rate of 60-day acute care hospitalization (mean [SD], 16.12% [3.70%] vs 15.42% [4.21%]), and a higher rate of having outpatient emergency department visits (mean [SD], 13.09% [3.98%] vs 12.39% [4.34%]) 60 days following an HHA admission. HHAs that changed ownership also served more Medicare beneficiaries (mean [SD], 538 [998] vs 291 [408]), but a lower percentage of beneficiaries who were dual eligible compared to HHAs that did not change ownership at baseline.

**Table 1.** Baseline Characteristics of Home Health Agencies That Changed Ownership and Matched Controls, 2016-2019^a^

Characteristic	Mean (SD)		*P* value^b^
	Matched control HHA (n = 2330)^c^^,^^d^	Ownership change HHA (n = 294)^d^	
Pretransaction ownership, No. (%)^e^			
For-profit	1899 (82)	235 (80)	.52
Nonprofit	305 (13)	41 (14)	.68
Government	126 (5)	18 (6)	.61
Any HHA branch, No. (%)^e^	451 (20)	69 (23)	.10
Operates hospice, No. (%)^e^	122 (5)	22 (7)	.11
Region, No. (%)^e^			
Northeast	172 (7)	23 (8)	.79
Midwest	393 (17)	46 (16)	.60
South	1482 (64)	189 (64)	.82
West	283 (12)	36 (12)	.96
Quality-of-care outcome			
Composite star ratings	3.24 (0.90)	3.35 (0.79)	.02
Timely initiation of care^f^	93.08 (7.25)	92.33 (6.96)	.07
Improvement in ambulation^f^	67.68 (14.06)	67.43 (10.83)	.69
Improvement in bed transferring^f^	63.71 (16.09)	64.23 (12.88)	.46
Improvement in bathing^f^	70.51 (15.36)	71.32 (11.78)	.22
Improvement in dyspnea^f^	65.01 (19.87)	68.21 (14.36)	.00
Improvement in management of oral medications^f^	55.95 (16.77)	57.33 (12.60)	.05
Rate of 60-d acute care hospitalization^f^	15.42 (4.21)	16.12 (3.70)	<.001
Rate of 60-d ED visits^f^	12.39 (4.34)	13.09 (3.98)	<.001
Size and capacity			
No. of Medicare beneficiaries	291 (408)	538 (998)	<.001
Per capita Medicare payment, $	5988 (2168)	5115 (1654)	<.001
Patient characteristics^g^			
Dual eligible	35.21 (23.60)	23.36 (13.49)	<.001
Rural	32.45 (37.27)	42.54 (38.41)	<.001
Racially and ethnically minoritized group^h^	30.46 (25.90)	20.87 (18.39)	<.001
Average HCC score	2.39 (0.47)	2.30 (0.40)	<.001
Staffing			
Registered nurse FTE	8.2 (61.58)	13.58 (39.14)	.03
LPN/LVN FTE	3.31 (16.57)	3.58 (9.96)	.66
Physical therapist FTE	2.32 (8.02)	4.08 (9.30)	<.001
Occupational therapist FTE	0.76 (1.96)	1.14 (2.24)	.02
Home health aide FTE	3.99 (19.74)	6.05 (25.51)	.23
Skilled nursing care, min/visit	46.17 (10.75)	46.98 (8.54)	.12
Physical therapy, min/visit	46.85 (8.78)	46.63 (7.20)	.61
Occupational therapy, min/visit	47.42 (9.01)	47.15 (8.09)	.59
Home health aide care, min/visit	55.78 (16.68)	56.13 (16.93)	.75

Abbreviations: ED, emergency department; FTE, full-time equivalent; HCC, hierarchical condition category; HHA, home health agency; LPN, licensed practical nurse; LVN, licensed vocational nurse.

^a^ The unit of analysis for quality-of-care outcomes is HHA quarters. The unit of analysis for patient characteristics, volumes, per capita payments, and staffing outcomes is HHA years.

^b^ *P* values were calculated from *t* tests of the difference in means between ownership change status at baseline; standard errors were clustered at the HHA level.

^c^ Up to 8 control HHAs (for-profit and nonprofit/public HHAs) that never changed ownership during the study period were matched to HHAs that changed ownership. All the variables used for matching were measured in the first observation year. This generated 2330 matched control HHAs (33 076 HHA quarters) for the 294 HHAs with a change of ownership from 2016 to 2019 (4767 HHA quarters).

^d^ Baseline characteristics were derived based on the pretransaction periods for HHAs that changed ownership and all time periods for matched control HHAs from 2016 to 2019.

^e^ Variables were used for matching. Reported values were measured in the first observation year.

^f^ Measures were presented as a percentage of home health episodes. Additional details can be found in eTable 3 in Supplement 1.

^g^ Measures were presented as a percentage of Medicare beneficiaries.

^h^ Race and ethnicity were recorded in the public use files based on beneficiaries’ most current Medicare records. Racially and ethnically minoritized groups were analyzed collectively, categorized as American Indian or Alaska Native, Asian and Pacific Islander, Black, Hispanic, other, and unknown, due to the high frequency of Centers for Medicare & Medicaid Services data suppression for services delivered to 10 or fewer beneficiaries.

**Figure 2.**
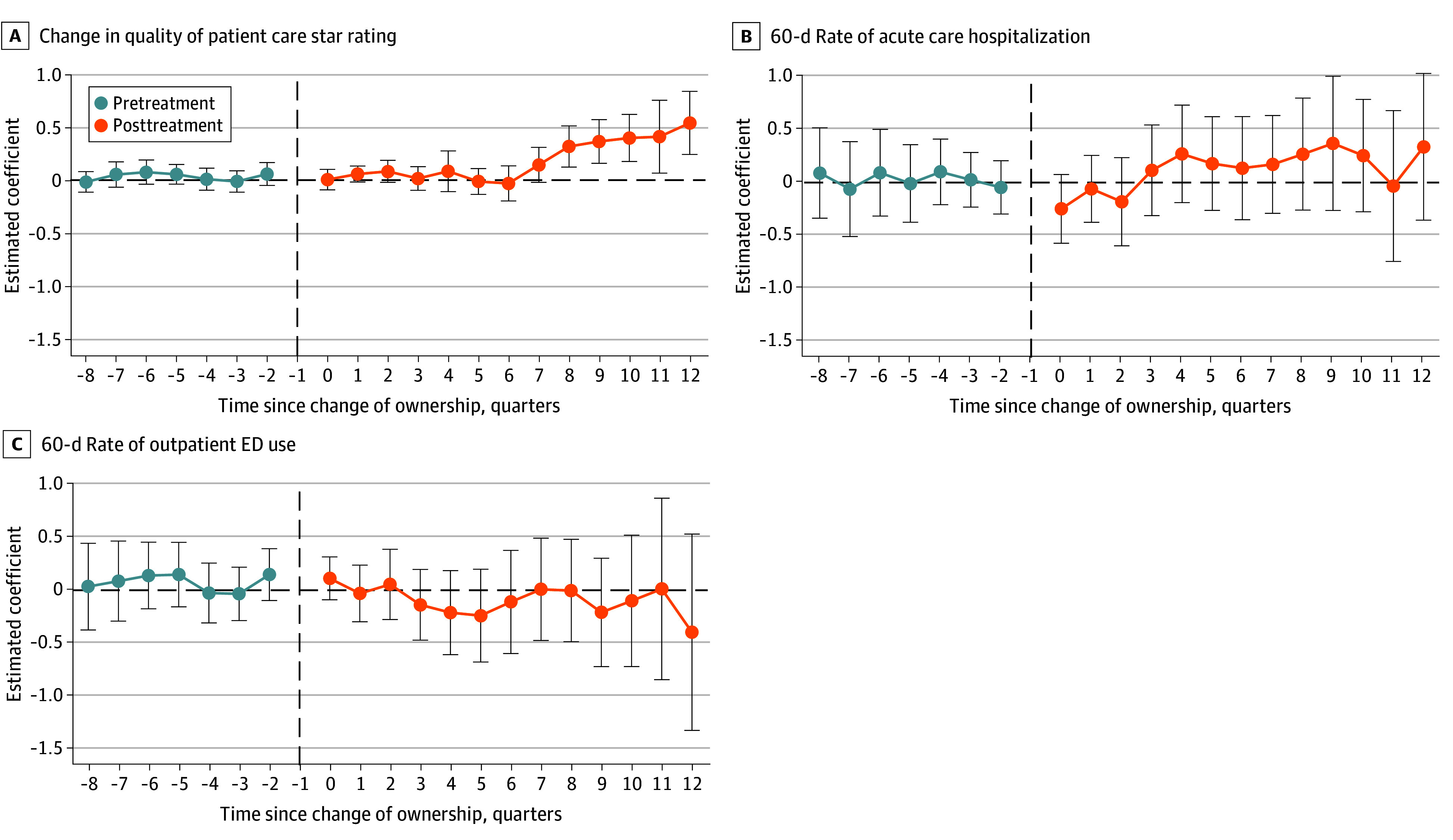
Quality-of-Care Outcomes for Home Health Agencies (HHAs) That Changed Ownership and Matched Controls HHA quarterly star ratings and claims-based quality metrics (ie, hospitalizations and emergency department [ED] visits) are presented in this figure. The line graphs show estimated coefficients (dots) and 95% CI (error bars) from the difference-in-differences event-study regression (N = 37 843). The unit of analysis is HHA quarters. The binary variable indicates whether HHAs changed ownership, interacting with 8 quarters before and 12 after the transaction. Up to 8 control HHAs were matched to those that changed ownership. Quarter 0 marks the transaction; quarter −1 was omitted. Regressions include HHA and year-quarter fixed effects. HHA quarters were weighted by patients enrolled in Medicare in the first study year. Standard errors were clustered at the HHA level. The vertical dashed lines represent the quarter before the transaction. Coefficient estimates were reported in eTable 5 in Supplement 1.

Figure 2 shows the estimated changes in quality outcomes at HHAs following ownership change from difference-in-differences with staggered timing models. In the 12-quarter (3-year) period after a change of ownership transaction, HHAs’ quarterly star ratings increased by 0.18 (95% CI, 0.05-0.31), compared to HHAs that did not change ownership (Table 2). The estimated difference increased in magnitude over time, showing a difference of 0.54 (standard error, 0.15) in the 12th quarter after the transaction (eTable 5 in Supplement 1). We did not observe significant improvement in 60-day rates of hospital admissions or outpatient emergency department visits.

**Table 2.** Outcomes for Home Health Agencies With Change of Ownership Transactions and Matched Controls by Transaction Types^a^

Outcome	All transaction types (n = 294)			For-profit to for-profit HHA (n = 237)			Nonprofit/public to for-profit HHA (n = 57)		
	Baseline mean (SD)	Estimated Δ (95% CI)^b^	Pretrend *P* value^c^	Baseline mean (SD)	Estimated Δ (95% CI)^b^	Pretrend *P* value^c^	Baseline mean (SD)	Estimated Δ (95% CI)^b^	Pretrend *P* value^c^
Quality of care, HHA quarters									
Quality-of-care star rating	3.62 (0.71)	0.18 (0.05 to 0.31)	.14	3.71 (0.74)	0.11 (−0.04 to 0.26)	.46	3.35 (0.53)	0.39 (0.18 to 0.59)	.16
Timely initiation of care, pp	92.70 (5.60)	0.33 (−0.30 to 0.96)	.01	93.67	−0.16 (−0.89 to 0.57)	.12	89.77 (6.06)	1.91 (0.84 to 2.98)	.002
Improvement in ambulation, pp	70.92 (8.92)	1.21 (−0.54 to 2.97)	.20	(5.09)	1.71 (−0.38 to 3.79)	.28	67.89 (5.99)	−0.77 (−3.52 to 1.99)	.17
Improvement in bed transferring, pp	67.94 (10.08)	0.64 (−1.62 to 2.90)	.30	71.91	0.88 (−1.87 to 3.63)	.77	67.05	−0.06 (−3.19 to 3.08)	.04
Improvement in bathing, pp	74.89 (8.99)	1.28 (−0.14 to 2.69)	.14	(9.48)	1.61 (−0.04 to 3.25)	.33	(7.65)	0.17 (−2.73 to 3.06)	.09
Improvement in dyspnea, pp	73.47 (10.74)	1.8 (−0.38 to 3.97)	.73	68.24 (10.75)	1.91 (−0.75 to 4.56)	.67	72.60 (6.17)	0.96 (−1.92 to 3.84)	.03
Improvement in management of oral medications, pp	62.06 (10.48)	1.86 (−0.25 to 3.96)	.48	75.65 (9.63)	2.36 (−0.14 to 4.86)	.44	73.46 (7.10)	−0.09 (−3.32 to 3.15)	.002
Rate of 60-d acute care hospitalization, pp	16.75 (2.57)	0.12 (−0.20 to 0.45)	.75	73.47 (11.70)	0.20 (−0.19 to 0.59)	.33	60.28 (7.02)	−0.15 (−0.67 to 0.36)	.76
Rate of 60-d outpatient ED use, pp	12.43 (2.64)	−0.10 (−0.44 to 0.24)	.30	62.64 (11.34)	−0.05 (−0.44 to 0.34)	.31	17.31 (2.60)	−0.26 (−0.93 to 0.4)	.56
Volume and per capita payment, HHA years^d^									
No. of Medicare beneficiaries	7.07 (1.30)	−0.02 (−0.11 to 0.07)	.15	6.97 (1.34)	0.04 (−0.06 to 0.13)	.97	7.35 (1.13)	−0.11 (−0.29 to 0.07)	.01
Medicare per capita payment	8.42 (0.26)	0.04 (−0.004 to 0.09)	.80	8.48 (0.25)	0.04 (−0.02 to 0.10)	.44	8.23 (0.18)	0.06 (−0.02 to 0.14)	.06
Staffing^d^									
Registered nurse FTE	3.07 (1.40)	−0.17 (−0.31 to −0.03)	.87	2.97 (1.46)	−0.14 (−0.26 to −0.01)	.29	3.36 (1.16)	−0.15 (−0.51 to 0.21)	.13
LPN/LVN FTE	1.44 (1.38)	−0.33 (−0.55 to −0.10)	.03	1.51 (1.47)	−0.46 (−0.65 to −0.26)	<.001	1.21 (0.97)	0.45 (−0.26 to 1.15)	.83
Physical therapist FTE	2.19 (1.30)	−0.1 (−0.27 to 0.06)	.20	2.16 (1.36)	−0.09 (−0.29 to 0.10)	.01	2.29 (1.05)	−0.06 (−0.35 to 0.24)	.24
Occupational therapist FTE	1.13 (1.20)	0.13 (−0.05 to 0.31)	.16	1.14 (1.25)	0.09 (−0.11 to 0.30)	.22	1.11 (1.01)	0.27 (−0.12 to 0.65)	.45
Home health aide FTE	1.44 (1.46)	−0.26 (−0.38 to −0.13)	.44	1.47 (1.60)	−0.26 (−0.41 to −0.11)	.22	1.35 (0.97)	−0.22 (−0.49 to 0.05)	.62
Skilled nursing care, min/visit	3.82 (0.19)	−0.05 (−0.09 to −0.01)	.51	3.80 (0.17)	−0.08 (−0.13 to −0.03)	.29	3.87 (0.24)	0.02 (−0.06 to 0.09)	.73
Physical therapy, min/visit	3.82 (0.17)	−0.03 (−0.05 to 0)	.68	3.81 (0.15)	−0.04 (−0.06 to −0.01)	.49	3.85 (0.22)	0 (−0.06 to 0.06)	.20
Occupational therapy, min/visit	3.82 (0.18)	−0.01 (−0.04 to 0.02)	.07	3.81 (0.16)	−0.02 (−0.05 to 0.01)	.04	3.86 (0.23)	0.02 (−0.07 to 0.10)	.33
Home health aide care, min/visit	3.93 (0.30)	−0.11 (−0.15 to −0.06)	.52	3.88 (0.24)	−0.10 (−0.14 to −0.05)	.95	4.06 (0.30)	−0.14 (−0.25 to −0.03)	<.001

Abbreviations: ED, emergency department; FTE, full-time equivalent; HHA, home health agency; LPN, licensed practical nurse; LVN, licensed vocational nurse; pp, percentage point.

^a^ Each observation (HHA quarters or HHA years) was weighted by the total number of unique patients served by Medicare in the first observation year of the study period. Pretreatment quarters differed for HHAs that changed ownership at different periods.

^b^ Estimates represent the dynamic aggregation of average treatment effects on the treated across 12 quarters posttransaction. Event-study coefficient estimates were reported in eTables 5 to 10 in Supplement 1.

^c^ Pretrend tests report the *P* value from a joint *F* test of the estimated coefficients pretransaction. Significant test results (*P* < 0.05) suggest evidence of pretrend.

^d^ Logarithmically transformed. For HHA year analyses, the observation was further limited with nonmissing outcomes for each regression.

**Figure 3.**
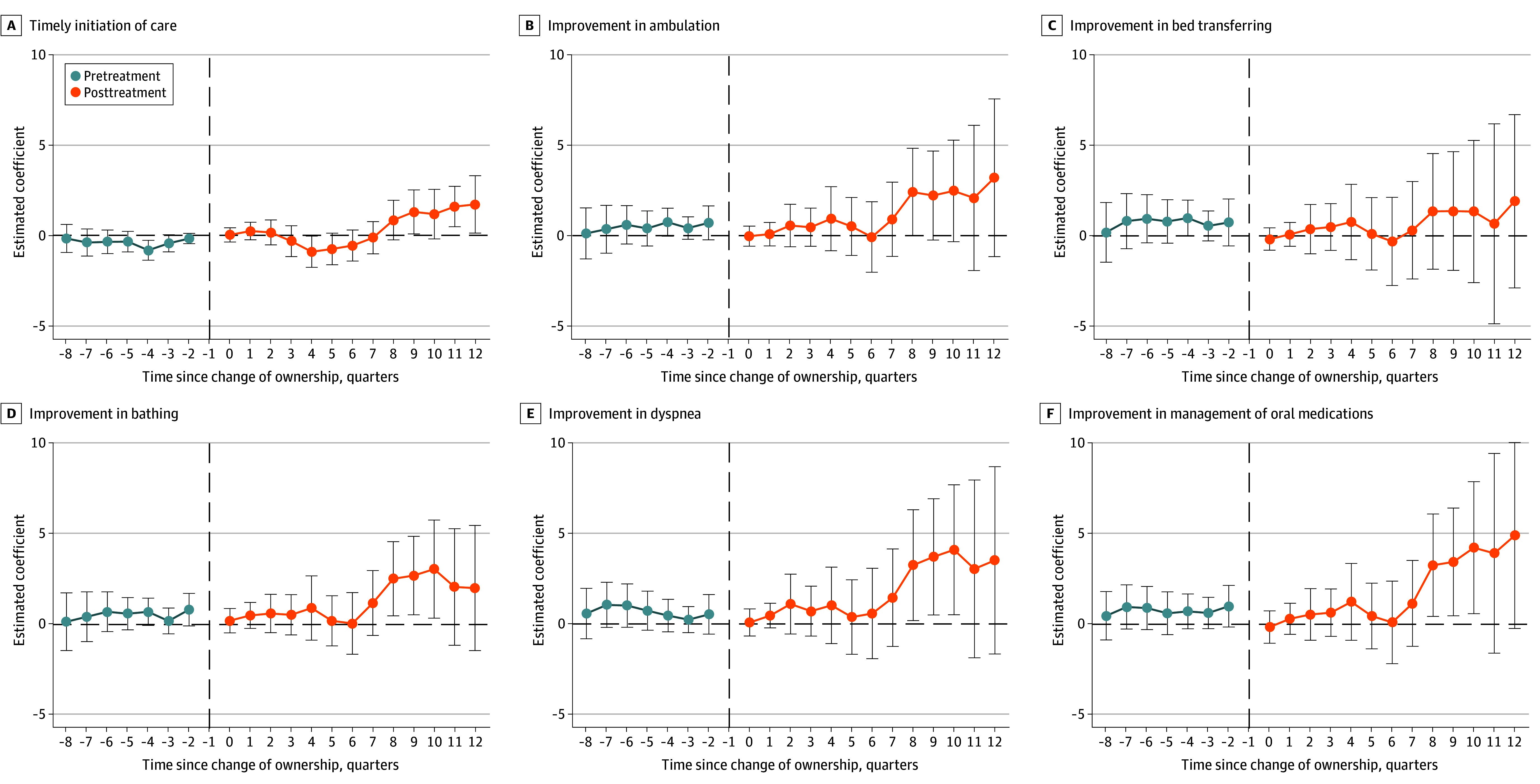
Outcome and Assessment Information Set (OASIS)–Based Quality of Care Outcomes for Home Health Agencies (HHAs) That Changed Ownership and Matched Controls Six process and outcome quality measures from the OASIS compose the overall star rating of an HHA. Each of these measures is presented in this figure. The line graphs display estimated coefficients (dots) and 95% CI (error bars) from the difference-in-differences event-study regression (N = 37 843). The unit of analysis is HHA quarters. The binary variable indicates HHA ownership changes, interacting with 8 quarters before and 12 after the transaction. Up to 8 control HHAs were matched. Quarter 0 marks the transaction; quarter −1 was omitted. Regressions include HHA and year-quarter fixed effects. HHA quarters were weighted by patients enrolled in Medicare in the first year. Standard errors were clustered at the HHA level. The vertical dashed lines represent the quarter before the transaction. Additional details may be found in eTable 3 in Supplement 1.

Figure 3 depicts changes in OASIS-based self-reported quality measures. Although the 3-year aggregated changes were not significant across measures (Table 2), significant increases were found in timely initiation of care, improvement in bathing, improvement in dyspnea, and improvement in management of oral medications starting from the eighth quarter after the transaction (eTable 5 in Supplement 1).

Table 2 reports difference-in-differences estimates for all outcomes overall and by transaction types. Although the aggregated change in the number of Medicare beneficiaries and per capita payment was not statistically significant, Medicare beneficiaries declined in the first year after the change of ownership, when analyzed annually, then increased to the pretransaction level in the subsequent years (eTable 6 in Supplement 1). We also found that Medicare per capita payment increased in the first 2 years after ownership change. When examining staffing levels, reductions were observed in full-time equivalents of registered nurses (−17% [95% CI, −31% to −3%]), licensed practice nurses/licensed vocational nurses (−33% [95% CI, −55% to −10%], with evidence of pretrend; *P* = .03), and home health aides (−26% [95% CI, −38% to −13%]) after ownership changes. We also observed reductions in per-visit minutes of skilled nursing care (−5% [95% CI, −9% to −1%]), physical therapy (−3% [95% CI, −5% to 0%]), and home health aide care (−11% [95% CI, −15% to −6%]). No significant changes were observed in full-time equivalents of occupational therapists.

We found qualitatively similar associations between ownership change and study outcomes when disaggregating by transaction types with several exceptions (Table 2; eTables 7-10 in Supplement 1). Increases in star ratings were only significant among transactions converting nonprofit HHAs to for-profit HHAs. Reductions in registered nurse, licensed practice nurse/licensed vocational nurse, and home health aide full-time equivalents, minutes per visit of skilled nursing care, and minutes per visit of physical therapy were only significant among transactions from for-profit to for-profit HHAs, possibly due to the small sample size of nonprofit to for-profit HHAs. In sensitivity analyses, most results were robust to unmatched controls (eTables 11-12 in Supplement 1) and outcomes without detrending adjustments (eTable 13 in Supplement 1).

## Discussion

This difference-in-differences analysis is the first, to our knowledge, to examine the association of ownership change with organizational characteristics and quality outcomes in the HHA setting. From January 2016 to December 2019, 429 HHAs changed ownership transactions, of which 60 converted from nonprofit or government-owned status to for-profit. These findings align with the growth of for-profit, chain-owned, and private equity–invested HHAs in recent decades.^7,12^

We found that 3 years after the transaction, ownership change was associated with almost a half-star increase in quality star ratings. However, the observed increase did not start until the seventh to eighth quarter after the transaction. The delayed increase may be due to the star rating measures’ typical 6-month to 9-month reporting lag.^21^ Consistent with the increased star ratings, we found increases in several self-reported OASIS-based measures used for calculating the overall star ratings approximately 8 quarters (2 years) after ownership change,^25^ including timely initiation of care, improvement in bathing, and improvement in management of oral medications. However, no changes were observed in the 60-day acute care hospitalization rates or outpatient emergency department visits. A possible explanation is that self-reported measures usually have a greater risk for upcoding than objective claims-based measures.^26,27,28^ Prior studies have shown that higher star ratings could help attract more patients and result in higher profits.^25^ Hence, our findings indicate that HHA buyers could more often focus on maximizing returns on investment, leading to upcoding for enhancing revenue streams without meaningful improvement in care quality. This is further supported by the findings that increases in star ratings and OASIS-based measures were more pronounced among HHAs that converted from nonprofit/public to for-profit organizations, relative to those that maintained for-profit status before and after the transaction.

We observed a decline in the number of Medicare beneficiaries in the year an HHA was changing ownership but an increase to pretransaction levels in subsequent years, indicating that ownership change could affect the short-term operations of HHAs. Meanwhile, we found increases in Medicare per capita payment in the first 2 years after ownership change. Prior research found for-profit agencies may be better at receiving higher reimbursement under the Medicare prospective payment system than nonprofit agencies, as they were more likely to shift resources toward more profitable therapy visits, reduce less profitable home health aide and medical social service visits, and recertify each patients’ episode.^11^ We found similar increases in per capita payments for nonprofit to for-profit and for-profit to for-profit transaction types, indicating that both could enable new owners to use similar strategies for-profit generation. Future research could explore the relationship between ownership change and the potential overutilization of certain HHA services.

The study results showed ownership change was associated with reduced staffing levels regardless of transaction type, including reductions in registered nurse, licensed practice nurse/licensed vocational nurse, and home health aide full-time equivalents, as well as per-visit minutes of skilled nursing care, physical therapy, and home health aide care. Staff reductions can lower operation costs, suggesting ownership changes at both nonprofit and for-profit HHAs could similarly shift the focus to profit generation; however, these changes were more pronounced at for-profit HHAs. To some extent, reduced staffing levels may indicate improved efficiency in providing care through economies of scale and the development of standardized care practices. However, the magnitude is alarming, with reductions in full-time equivalents of several types of professionals by more than 20% accompanied by reduced per-visit minutes. As substantial evidence links staffing levels to quality of care in other health care settings,^29,30,31^ the findings of our study reveal that HHAs’ ownership change could adversely impact quality of care. Although we did not observe diminished OASIS-based or claim-based quality measures, implications for patient outcomes and experiences remain unclear. These results have implications for understanding the quality of care through the lens of organizational ownership. Quality considerations in antitrust enforcement may substantially affect operations and care processes across organizations with different ownership structures.

### Limitations

The study results should be interpreted while considering several limitations. First, although the CMS Change of Ownership data have improved the financial and ownership transparency of health care organizations,^32^ the quality and accuracy of the data merit further auditing and cross-validation.^33^ Our analysis found considerable discrepancies between the current change of ownership and Provider of Services files on how transactions were documented. For example, 20% of ownership change transactions identified in the current Change of Ownership file were not captured in the linked Provider of Services from 2016 to 2019. Second, our analyses relied on publicly available files via CMS, including Home Health Compare, Provider of Services, and HHA Utilization and Payment Public Use File. Due to different reporting requirements and data suppression rules, our analyses can only be generalized to HHAs with reported star ratings and claims-based quality measures. More granular assessment using OASIS and claims is needed. Third, even among for-profit HHAs, organizations may still have different ownership structures (eg, corporate, limited liability company, private equity involvement) that could provide differing incentives. As CMS moves to capture more granular information in ownership data,^33^ future studies are needed to examine how much private equity and other investors are involved in HHA ownership change and the implications for patient care.

## Conclusions

In this difference-in-differences analysis comparing HHAs that changed ownership with matched controls, ownership change was associated with higher star ratings but not with changes in claim-based quality measures. More pronounced increases in star ratings were observed among nonprofit/public HHAs that converted to for-profit organizations, suggesting ownership change may change HHAs’ focus toward maximizing returns on investments. Ownership change was associated with higher Medicare per capita payments. We also observed staff reductions regardless of transaction type, raising concerns about quality of care.

## Notes

Supplement 1. eMethods. Supplemental Data Source Description and Empirical Approach eFigure 1. Schematic of Home Health Agency Change of Ownership Transaction eFigure 2. Distribution of the Timing of Home Health Agency Change of Ownership Transactions eTable 1. Sample Selection for HHA Quarter–Level Analyses eTable 2. Sample Selection for HHA Year–Level Analyses eTable 3. Outcome Definitions and Data Sources eTable 4. Reporting Period for Home Health Quality of Patient Care Star Ratings and Claims-based Measures eTable 5. Quality of Care Outcomes for HHAs With Change of Ownership Transactions and Matched Controls (All Transactions) eTable 6. Patient Volumes, Per capita Payment, and Staffing Outcomes for HHAs with Change of Ownership Transactions and Matched Controls (All Transactions) eTable 7. Quality of Care Outcomes for HHAs with Change of Ownership Transactions and Matched Controls (For-Profit to For-Profit Transactions) eTable 8. Patient Volumes, Per capita Payment, and Staffing Outcomes for HHAs with Change of Ownership Transactions and Matched Controls (For-Profit to For-Profit Transactions) eTable 9. Quality of Care Outcomes for HHAs with Change of Ownership Transactions and Matched Controls (Nonprofit/Public to For-Profit Transactions) eTable 10. Patient Volumes, Per capita Payment, and Staffing Outcomes for HHAs with Change of Ownership Transactions and Matched Controls (Nonprofit/Public to For-Profit Transactions) eTable 11. Baseline Characteristics of HHAs with Change of Ownership Transactions and Unmatched Controls eTable 12. Outcomes for HHAs with Change of Ownership Transactions and Unmatched Controls, by Transaction Types eTable 13. Outcomes for HHAs with Change of Ownership Transactions and Matched Controls, by Transaction Types (without Detrending) eReferences

Supplement 2. Data Sharing Statement
