# Supplementary material for: Change of Ownership and Quality of Home Health Agency Care
Source: JAMA Health Forum. 2024 Nov 1;5(11):e243767. doi: 10.1001/jamahealthforum.2024.3767 (PMC11530943; doi:10.1001/jamahealthforum.2024.3767)
Supplement: Supplement 1. — eMethods. Supplemental Data Source Description and Empirical Approach eFigure 1. Schematic of Home Health Agency Change of Ownership Transaction eFigure 2. Distribution of the Timing of Home Health Agency Change of Ownership Transactions eTable 1. Sample Selection for HHA Quarter–Level Analyses eTable 2. Sample Selection for HHA Year–Level Analyses eTable 3. Outcome Definitions and Data Sources eTable 4. Reporting Period for Home Health Quality of Patient Care Star Ratings and Claims-based Measures eTable 5. Quality of Care Outcomes for HHAs With Change of Ownership Transactions and Matched Controls (All Transactions) eTable 6. Patient Volumes, Per capita Payment, and Staffing Outcomes for HHAs with Change of Ownership Transactions and Matched Controls (All Transactions) eTable 7. Quality of Care Outcomes for HHAs with Change of Ownership Transactions and Matched Controls (For-Profit to For-Profit Transactions) eTable 8. Patient Volumes, Per capita Payment, and Staffing Outcomes for HHAs with Change of Ownership Transactions and Matched Controls (For-Profit to For-Profit Transactions) eTable 9. Quality of Care Outcomes for HHAs with Change of Ownership Transactions and Matched Controls (Nonprofit/Public to For-Profit Transactions) eTable 10. Patient Volumes, Per capita Payment, and Staffing Outcomes for HHAs with Change of Ownership Transactions and Matched Controls (Nonprofit/Public to For-Profit Transactions) eTable 11. Baseline Characteristics of HHAs with Change of Ownership Transactions and Unmatched Controls eTable 12. Outcomes for HHAs with Change of Ownership Transactions and Unmatched Controls, by Transaction Types eTable 13. Outcomes for HHAs with Change of Ownership Transactions and Matched Controls, by Transaction Types (without Detrending) eReferences [file jamahealthforum-e243767-s001.pdf]

Supplemental Online Content

Zhang Z, Li K, Wang S, Fashaw-Walters S, Hou Y. Change of ownership and quality of home health agency care. *JAMA Health Forum.* Published online November 1, 2024.
doi:10.1001/jamahealthforum.2024.3767

eMethods. Supplemental Data Source Description and Empirical Approach

eFigure 1. Schematic of Home Health Agency Change of Ownership Transaction

eFigure 2. Distribution of the Timing of Home Health Agency Change of Ownership Transactions **eTable 1.**

Sample Selection for HHA-Quarter–Level Analyses

eTable 2. Sample Selection for HHA-Year–Level Analyses

eTable 3. Outcome Definitions and Data Sources

eTable 4. Reporting Period for Home Health Quality of Patient Care Star Ratings and Claims-based Measures

eTable 5. Quality of Care Outcomes for HHAs with Change of Ownership Transactions and Matched Controls (All Transactions)

eTable 6. Patient Volumes, Per-capita Payment, and Staffing Outcomes for HHAs with Change of Ownership Transactions and Matched Controls (All Transactions)

eTable 7. Quality of Care Outcomes for HHAs with Change of Ownership Transactions and Matched Controls (FP-FP Transactions)

eTable 8. Patient Volumes, Per-capita Payment, and Staffing Outcomes for HHAs with Change of Ownership Transactions and Matched Controls (FP-FP Transactions)

eTable 9. Quality of Care Outcomes for HHAs with Change of Ownership Transactions and Matched Controls (NFP-FP Transactions)

eTable 10. Patient Volumes, Per-capita Payment, and Staffing Outcomes for HHAs with Change of Ownership Transactions and Matched Controls (NFP-FP Transactions)

eTable 11. Baseline Characteristics of HHAs with Change of Ownership Transactions and Unmatched Controls

eTable 12. Outcomes for HHAs with Change of Ownership Transactions and Unmatched Controls, by Transaction Types

eTable 13. Outcomes for HHAs with Change of Ownership Transactions and Matched Controls, by Transaction Types (without Detrending)

eReferences

This supplemental material has been provided by the authors to give readers additional information about their work.

© 2024 Zhang Z et al. *JAMA Health Forum.*

eMethods

1. Supplemental data source description

Home Health Compare (HHC). The Centers for Medicare and Medicaid Services (CMS) HHC file contains a list of Medicare-certified Home Health Agencies (HHA) and quarterly updated quality of care measures required by home health public reporting.[1] We included all HHAs included in this file to assess eligibility and merged them with the CHOW file. We obtained HHA's quality of patient care star ratings and two claims-based quality measures from this file. HHC files were updated quarterly.

Provider of Service (POS) File. The POS file contains provider-level characteristics for Medicare-certified hospitals and other facilities, including HHAs.[2] We obtained HHA's ownership status (categorized as for-profit, nonprofit, or government-owned), staffing full-time equivalents (FTE) by health care professionals, and the state where the HHA is located. POS files were updated annually prior to 2019.

The Medicare HHA Utilization and Payment PUF. The HHA Utilization and Payment PUF contains provider-level information on service utilization, payment, patient demographics, and clinical characteristics by HHAs annually.[3] We obtained the number of unique beneficiaries, total HHA Medicare payment amount, percent of Medicare-Medicaid dually eligible beneficiaries, percent of Medicare beneficiaries in a rural ZIP code, percent of beneficiaries who are White, average hierarchical condition category (HCC) scores, visit counts by services (skilled nursing, physical therapy, occupational therapy, and home health aide), and minutes by services (skilled nursing, PT, OT, and home health aide) from the HHA PUF. The HHA PUF files were updated annually.

© 2024 Zhang Z et al. JAMA Health Forum.

2. Empirical approach

For each HHA-quarter-level outcome, we estimated the following difference-in-differences (DD) event-study specification:

$$Y_{it} = \beta_0 + Treated_{it} * [\sum_{j=-8}^{-2} \boldsymbol{\beta_j} 1\{t - t_k^* = j\} + \sum_{j=0}^{12} \boldsymbol{\delta_j} 1\{t - t_k^* = j\}] + HHA_i + YearQuarter_t + \varepsilon_{it} \quad (1)$$

The event-study specification for HHA-year-level outcomes was defined analogously:

$$Y_{it} = \beta_0 + Treated_{it} * [\sum_{j=-4}^{-2} \boldsymbol{\beta_j} 1\{t - t_k^* = j\} + \sum_{j=0}^{3} \boldsymbol{\delta_j} 1\{t - t_k^* = j\}] + HHA_i + Year_t + \varepsilon_{it} \quad (2)$$

where Y_{it} is the outcome of interest (quality of care, patient volume and per-capita payment, and staffing measures) for HHA i in calendar year quarter (or calendar year) t. We included the HHA fixed effects (HHA_i) to account for pre-transaction time-invariant differences (including time-invariant state-level differences because HHAs are perfectly nested within States) between HHAs that changed ownership and matched control HHAs that did not. The quarter-year or year-fixed effects ($YearQuarter_t$ and $Year_t$) adjusted for the year-by-quarter or year trends, respectively, in outcomes for the matched control group. δ_j are the coefficient of interest, capturing the average treatment effects of CHOW on outcomes on the treated HHAs. Standard errors were clustered at the HHA level.

Callaway and Sant'Anna Approach. We used the Callaway and Sant'Anna approach to account for the staggered occurrence of change of ownership transactions. We followed the recently recommended approach to correct the asymmetric construction of pre- and post-treatment coefficients under the Callaway and Sant'Anna approach.[4] Specifically, we estimated staggered difference-in-difference regressions using the Stata command *csdid2* with the recommended option *long2*.

Weighting. All regressions were weighted by Medicare patients served during the first observation year of the study period to account for the number of beneficiaries contributing to the HHA-level outcomes and, in turn, to reduce the risk of overweighting smaller HHAs.

© 2024 Zhang Z et al. JAMA Health Forum.

Matching. We matched each HHA that underwent CHOW to up to 8 control HHAs (including for-profit, nonprofit, and public HHAs) that never had ownership transactions during the study period. Following the existing literature,[5] we first used exact matching based on year, ownership type (for-profit vs. nonprofit or public), US census region (Northeast, Midwest, South, West), whether the agency has any branches, and whether the agency also participates in the Medicare program as a hospice. Nearest neighbor matching was further used for the HHA size (total number of Medicare beneficiaries); up to 8 nearest neighbors were kept in the analyses. All the variables used for matching were measured in the first observation year.

Testing for Pre-trends. We assessed the validity of the DD parallel trend assumption by testing the presence of differential pre-trends using a joint F-test. We tested and reported whether the estimated coefficients for up to 5 quarters or 4 years pre-transaction (β_j) are equal and not statistically significant from zero; a significant test result ($p<0.05$) suggests evidence of pre-trend.

Detrending. To minimize any remaining differential pre-trends in outcomes between HHAs with CHOW and matched controls, we followed the existing literature to detrend the outcome variables.[6–8] Specifically, we estimated a differential linear pre-trend for each outcome variable using the data from the pre-transaction period:

$$Y_{it} = \beta_0 + \boldsymbol{\alpha} * Treated_{it} * Timeline_t + HHA_i + YearQuarter_t + \varepsilon_{it} \quad (3)$$

We then residualized the outcome variables for all year-quarters by removing the estimated pre-trend:

$$\widehat{Y_{it}} = Y_{it} - \widehat{\boldsymbol{\alpha}} * Treated_{it} * Timeline_t \quad (4)$$

We then use $\widehat{Y_{it}}$ as the outcome and estimated the equations (1). Similar approaches were used for the HHA-year analyses. This process allows us to remove the linear pre-trend from the post-transaction period.

© 2024 Zhang Z et al. JAMA Health Forum.

Sensitivity Analyses. We provided two sets of sensitivity analyses: (1) DD using unmatched samples and (2) Matched DD analyses without detrending.

© 2024 Zhang Z et al. JAMA Health Forum.

eFigure 1. Schematic of Home Health Agency Change of Ownership Transaction

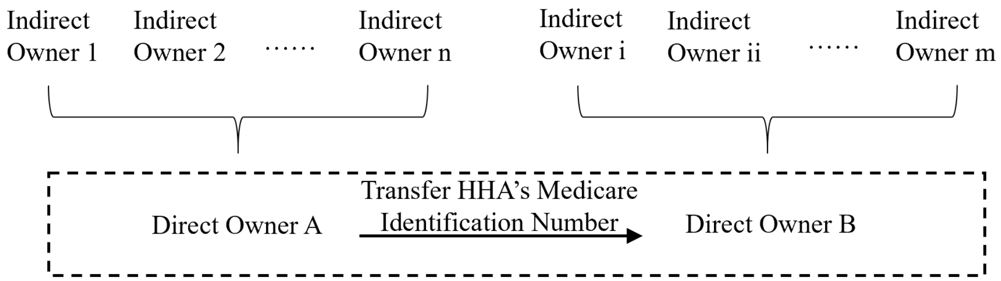

Notes: eFigure 1 illustrates a schematic of the HHA CHOW Transaction, defined as the Medicare identification number of an acquired HHA transferred from the seller (the previous owner) to the buyer (the new owner). Each HHA was identified using the CCN. According to the CMS data guidance, the direct owner is defined as the individual or organization that possesses the equity in the capital, the stock, or the profits of the disclosing entity.[9] Each direct owner may themselves be owned by other organizations or individuals, which were considered indirect owners. The CHOW ownership data do not identify the ultimate owner among all indirect owners (if any) unless the ultimate (indirect) owner is an individual.[10]
Abbreviations: HHA, home health agency; CHOW, change of ownership; CCN, Centers for Medicare and Medicaid Services Certification Numbers.

© 2024 Zhang Z et al. JAMA Health Forum.

eFigure 2. Distribution of the Timing of Home Health Agency Change of Ownership Transactions

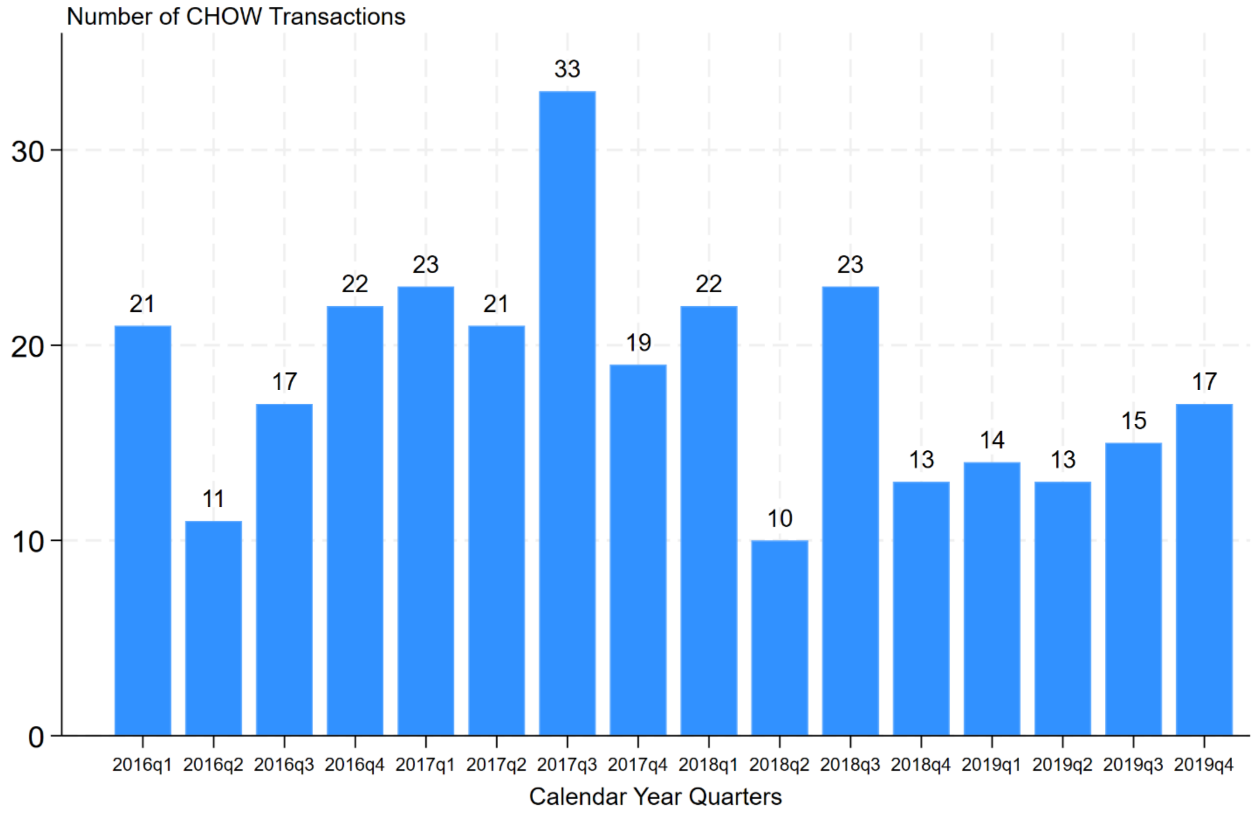

Notes: eFigure 2 shows the full distribution of the timing of HHA CHOW transactions (n=294) included in the analytic sample from 2016 quarter 1 to 2019 quarter 4.
Abbreviations: HHA, home health agency; CHOW, change of ownership.

© 2024 Zhang Z et al. JAMA Health Forum.

eTable 1. Sample Selection for HHA-Quarter–Level Analyses

	Sample Selection Steps	HHAs	HHA-quarters	Treated HHAs	Control HHAs
1	Start: HHA-quarters from Medicare-certified HHAs from 2015 Q3 to 2019 Q4 linked to the CHOW file	15,068	271,224	664	14,404
2	Drop if HHAs underwent CHOW that resulted in CCN dissolution	15,057	271,026	653	14,404
3	Drop if HHAs underwent CHOW in 2020 Q1-2022 Q4 (later treated)	14,833	266,994	429	14,404
4	Drop if HHAs underwent NFP-NFP or FP-NFP CHOW	14,753	265,554	349	14,404
5	Drop if HHAs in the control group with non-zero CHOW numbers documented in the POS file	12,540	223,004	349	12,191
6	Drop if missing any of the eight quality-of-care outcomes	8,063	105,774	326	7,737
7	Drop if missing all of the secondary outcomes	8,063	105,744	326	7,737
8	Drop if missing analytic weights	7,849	104,933	320	7,529
9	Drop if HHA-quarters (years) that were singletons	7,473	104,557	310	7,163
10	Drop if treated HHAs without pre-CHOW data after listwise deletion of missing data	7,457	104,487	294	7,163
11	Matched control sample	2,624	37,843	294	2,330

Abbreviations: HHA, home health agency; CHOW, change of ownership; CCN, Centers for Medicare and Medicaid Services Certification Numbers; POS, provider of service; NFP, non-profit or government-owned; FP, for-profit.

© 2024 Zhang Z et al. JAMA Health Forum.

eTable 2. Sample Selection for HHA-Year–Level Analyses

	Sample Selection Steps	HHAs	HHA-years	Treated HHAs	Control HHAs
1	Start: HHA-quarters from Medicare-certified HHAs from 2015 Q3 to 2019 Q4 linked to the CHOW file	15,068	75,340	664	14,404
2	Drop if HHAs underwent CHOW that resulted in CCN dissolution	15,057	75,285	653	14,404
3	Drop if HHAs underwent CHOW in 2020 Q1-2022 Q4 (later treated)	14,833	74,165	429	14,404
4	Drop if HHAs underwent NFP-NFP or FP-NFP CHOW	14,753	73,765	349	14,404
5	Drop if HHAs in the control group with non-zero CHOW numbers documented in the POS file	12,540	62,021	349	12,191
6	Drop if missing any of the eight quality-of-care outcomes	8,063	32,451	326	7,737
7	Drop if missing all of the secondary outcomes	8,063	32,421	326	7,737
8	Drop if missing analytic weights	7,849	32,027	320	7,529
9	Drop if HHA-years that were singletons	7,117	31,295	297	6,820
10	Drop if treated HHAs without pre-CHOW data after listwise deletion of missing data	7,108	31,264	288	6,820
11	Matched control sample	2,514	11,288	288	2,226

Abbreviations: HHA, home health agency; CHOW, change of ownership; CCN, Centers for Medicare and Medicaid Services Certification Numbers; POS, provider of service; NFP, non-profit or government-owned; FP, for-profit.

© 2024 Zhang Z et al. JAMA Health Forum.

eTable 3. Outcome Definitions and Data Sources

Outcome Measure	Definition	Source
**Quality of Care**		
Star ratings	Composite quality of care star ratings	HHC
Timely Initiation of Care	Percentage of home health episodes initiated within 2 days of the referral date or inpatient discharge date.	HHC
Improvement in Ambulation	Percentage of home health episodes during which the patient improved in ability to ambulate.	HHC
Improvement in Bed Transferring	Percentage of home health episodes during which the patient improved in ability to get in and out of bed.	HHC
Improvement in Bathing	Percentage of home health episodes during which the patient improved in bathing self.	HHC
Improvement in Dyspnea	Percentage of home health episodes during which the patient became less short of breath or dyspneic.	HHC
Improvement in Management of Oral Medications	Percentage of home health episodes during which the patient improved in ability to take oral medications correctly.	HHC
Rate of 60-Day Acute Care Hospitalization	Percentage of home health stays in which patients were admitted to an acute care hospital during the 60 days following the start of the home health stay.	HHC
Rate of 60-Day ED Use without Hospitalization	Percentage of home health stays in which patients had ED visits but were not admitted to the hospital during the 60 days following the start of the home health stay.	HHC
**Size and Capacity**		
Number of Medicare beneficiaries (logged)	Number of Medicare beneficiaries	HHA PUF
Medicare per-capita payment (logged)	Calculated as an HHA's total Medicare payment divided by the total number of beneficiaries served each year	HHA PUF
**Patient Characteristics**		
Dually eligible beneficiaries	Percentage of Medicare-Medicaid dually eligible	HHA PUF
Rural beneficiaries	Percentage of Medicare beneficiaries with rural ZIP codes	HHA PUF
Racial and ethnic minoritized beneficiaries	Percentage of Medicare beneficiaries who are American Indian or Alaska Native, Asian, Black, Hispanic, Other, and Unknown	HHA PUF
Average HCC risk scores	Average HCC risk scores	HHA PUF
**Staffing**		
FTE by type of healthcare professionals	Include registered nurse, LPN/LVN, PT, OT, and home health aide	POS
Minutes per visit by service types	Calculated as total minutes by each type of service (skilled nursing, PT, OT, and home health aide) divided by the total number of each type of visit.	HHA PUF

Abbreviations: HHA, home health agency; CHOW, change of ownership; ED, emergency department; HCC, hierarchical condition category; FTE, full-time equivalent; PT, physical therapy; OT, occupational therapy; LPN, licensed practical nurse; LVN, licensed vocational nurse; HHC, home health compare; POS, provider of service; PUF, public use file.

eTable 4. Reporting Period for Home Health Quality of Care Star Ratings and Claims-based Measures

Difference-in-Difference study period	OASIS-based process measures[a, c]	Claims-based quality measures[b, c]
2015Q3	2014Q1-2014Q4	2013Q4-2014Q3
2015Q4	2014Q2-2015Q1	2014Q1-2014Q4
2016Q1	2014Q3-2015Q2	2014Q2-2015Q1
2016Q2	2014Q4-2015Q3	2014Q3-2015Q2
2016Q3	2015Q1-2015Q4	2014Q4-2015Q3
2016Q4	2015Q2-2016Q1	2015Q1-2015Q4
2017Q1	2015Q3-2016Q2	2015Q2-2016Q1
2017Q2	2015Q4-2016Q3	2015Q3-2016Q2
2017Q3	2016Q1-2016Q4	2015Q4-2016Q3
2017Q4[d]	2016Q1-2016Q4	2016Q1-2016Q4
2018Q1	2016Q2-2017Q1	2016Q2-2017Q1
2018Q2	2016Q3-2017Q2	2016Q3-2017Q2
2018Q3	2016Q4-2017Q3	2016Q4-2017Q3
2018Q4	2017Q1-2017Q4	2017Q1-2017Q4
2019Q1	2017Q2-2018Q1	2017Q1-2017Q4
2019Q2[e]	2017Q3-2018Q2	2017Q1-2017Q4
2019Q3	2017Q4-2018Q3	2017Q1-2017Q4
2019Q4	2018Q1-2018Q4	2017Q1-2017Q4

Notes: [a]OASIS-based quality measures include timely initiation of care, improvement in ambulation, improvement in bed transferring, improvement in bathing, improvement in dyspnea, and improvement in the management of oral medications.[1]
[b] Claims-based quality measures include the percentage of patients admitted to acute care hospitals within 60 days following a home health admission.
[c] The overall star rating was calculated by averaging across 6 OASIS-based process measures and 1 claims-based quality measure (acute care hospitalization).
[d] Reporting for OASIS-based quality measures moved from a 6- to a 9-month lag.
[e] Claims-based quality measures reporting moved to annual updates.
Abbreviations: OASIS, the Outcome and Assessment Information Set.

© 2024 Zhang Z et al. JAMA Health Forum.

eTable 5. Quality of Care Outcomes for HHAs with Change of Ownership Transactions and Matched Controls (All Transactions)

Outcome	Composite Star Ratings	Timely Initiation of care	Improvement in Ambulation	Improvement in Bed Transferring	Improvement in Bathing
	Coefficient (SE)	Coefficient (SE)	Coefficient (SE)	Coefficient (SE)	Coefficient (SE)
Pre Average	0.03	-0.39	0.48	0.72	0.51
	(0.04)	(0.24)	(0.40)	(0.49)	(0.42)
Post Average	0.18***	0.33	1.21	0.64	1.28*
	(0.07)	(0.32)	(0.90)	(1.15)	(0.72)
Time Period					
-5	0.05	-0.35	0.40	0.80	0.60
	(0.05)	(0.29)	(0.50)	(0.61)	(0.46)
-4	0.01	-0.83***	0.75*	0.99*	0.70*
	(0.05)	(0.28)	(0.39)	(0.50)	(0.39)
-3	-0.01	-0.44*	0.42	0.55	0.19
	(0.05)	(0.24)	(0.32)	(0.42)	(0.36)
-2	0.06	-0.18	0.71	0.74	0.82*
	(0.06)	(0.15)	(0.48)	(0.66)	(0.46)
0	0.00	0.02	-0.03	-0.18	0.21
	(0.05)	(0.20)	(0.28)	(0.32)	(0.34)
1	0.06	0.23	0.09	0.08	0.50
	(0.04)	(0.25)	(0.33)	(0.34)	(0.36)
4	0.08	-0.91**	0.94	0.77	0.91
	(0.10)	(0.44)	(0.90)	(1.06)	(0.91)
8	0.32***	0.84	2.41*	1.35	2.53**
	(0.10)	(0.56)	(1.24)	(1.63)	(1.05)
12	0.54***	1.71**	3.20	1.91	2.01
	(0.15)	(0.81)	(2.23)	(2.44)	(1.77)
Observations	37843	37843	37843	37843	37843
Outcome	Improvement in Dyspnea	Improvement in Management of Oral Medication	60-day Acute Care Hospitalization	60-day Outpatient ED Use	
	Coefficient (SE)	Coefficient (SE)	Coefficient (SE)	Coefficient (SE)	
Pre Average	0.66	0.76*	0.03	0.07	
	(0.43)	(0.44)	(0.14)	(0.12)	
Post Average	1.80	1.86*	0.12	-0.10	
	(1.11)	(1.07)	(0.17)	(0.17)	
Time period					
-5	0.73	0.61	-0.01	0.15	
	(0.55)	(0.60)	(0.19)	(0.15)	
-4	0.46	0.72	0.10	-0.03	
	(0.46)	(0.49)	(0.16)	(0.14)	
-3	0.24	0.63	0.03	-0.03	
	(0.37)	(0.44)	(0.13)	(0.13)	
-2	0.53	1.00*	-0.05	0.15	
	(0.56)	(0.59)	(0.13)	(0.12)	
0	0.08	-0.15	-0.25	0.11	
	(0.38)	(0.46)	(0.17)	(0.10)	
1	0.46	0.31	-0.06	-0.03	
	(0.35)	(0.44)	(0.16)	(0.14)	
4	1.03	1.24	0.27	-0.21	
	(1.08)	(1.09)	(0.23)	(0.20)	
8	3.25**	3.27**	0.27	-0.00	
	(1.56)	(1.44)	(0.27)	(0.25)	
12	3.51	4.91*	0.34	-0.40	
	(2.64)	(2.62)	(0.35)	(0.47)	
Observations	37843	37843	37843	37843	

Notes: eTable 5 shows the full set of coefficients estimated from the event-study specification (selected time periods were reported for simplicity; full results available upon request). The unit of analysis is HHA-quarters. “All Transactions” indicate all types of HHA CHOW transactions, including both changes within the for-profit status and changes from nonprofit or public to for-profit status, compared with all matched control HHAs (for-profit, nonprofit,

© 2024 Zhang Z et al. JAMA Health Forum.

and public) that never had CHOW during the study period. Pre-average is the dynamic aggregation of average treatment effects on the treated across 8 quarters before the transaction. Post-average is the dynamic aggregation of average treatment effects on the treated across 12 quarters following the transaction. ***p<0.001, ** p<0.05, * p<0.1.

Abbreviations: HHA, home health agency; CHOW, change of ownership; ED, emergency department.

© 2024 Zhang Z et al. JAMA Health Forum.

eTable 6. Patient Volumes, Per-capita Payment, and Staffing Outcomes for HHAs with Change of Ownership Transactions and Matched Controls (All Transactions)

Outcome	Medicare Beneficiaries	Per-capita Payment	Registered Nurse FTE	LPN/LVN FTE	Physical Therapy FTE	Occupational Therapy FTE
	Coefficient (SE)	Coefficient (SE)	Coefficient (SE)	Coefficient (SE)	Coefficient (SE)	Coefficient (SE)
Pre Average	0.03	0.01	0.02	0.10	0.03	0.04
	(0.04)	(0.01)	(0.04)	(0.13)	(0.09)	(0.07)
Post Average	-0.02	0.04*	-0.17**	-0.33***	-0.10	0.13
	(0.05)	(0.02)	(0.07)	(0.12)	(0.08)	(0.09)
Time Period						
-4	0.03	0.01	0.03	-0.13	-0.06	0.08
	(0.07)	(0.01)	(0.07)	(0.21)	(0.22)	(0.16)
-3	0.03	0.01	0.04	0.21	0.12*	0.09
	(0.05)	(0.01)	(0.05)	(0.21)	(0.07)	(0.06)
-2	0.04*	0.00	0.00	0.21***	0.02	-0.04
	(0.02)	(0.01)	(0.03)	(0.08)	(0.03)	(0.04)
0	-0.13***	0.01	-0.10**	0.06	-0.09**	0.12
	(0.04)	(0.01)	(0.05)	(0.11)	(0.04)	(0.07)
1	-0.02	0.07***	-0.20***	-0.21*	-0.15	0.11
	(0.05)	(0.02)	(0.07)	(0.11)	(0.10)	(0.09)
2	0.05	0.08***	-0.15**	-0.37**	-0.15	0.15
	(0.05)	(0.03)	(0.08)	(0.15)	(0.11)	(0.12)
3	0.02	0.03	-0.22	-0.79***	-0.02	0.15
	(0.11)	(0.05)	(0.18)	(0.25)	(0.18)	(0.22)
Observations[b]	10934	10934	10892	8388	6745	5086
Outcome	Home Health Aide FTE	Minutes per Skilled Nursing Visit	Minutes per Physical Therapy Visit	Minutes per Occupational Therapy Visit	Minutes Per Home Health Aide Visit	
	Coefficient (SE)	Coefficient (SE)	Coefficient (SE)	Coefficient (SE)	Coefficient (SE)	
Pre Average	0.00	0.01	0.01	0.01	-0.00	
	(0.06)	(0.02)	(0.01)	(0.01)	(0.01)	
Post Average	-0.26***	-0.05**	-0.03**	-0.01	-0.11***	
	(0.07)	(0.02)	(0.01)	(0.01)	(0.02)	
Time period						
-4	-0.10	0.03	0.01	0.01	0.01	
	(0.10)	(0.03)	(0.02)	(0.03)	(0.03)	
-3	0.09	0.01	0.01	0.02*	-0.01	
	(0.10)	(0.02)	(0.01)	(0.01)	(0.01)	
-2	0.02	-0.01	-0.00	0.00	-0.02	
	(0.04)	(0.01)	(0.01)	(0.01)	(0.01)	
0	-0.06	-0.02	-0.01	-0.00	-0.05**	
	(0.04)	(0.02)	(0.01)	(0.01)	(0.02)	
1	-0.12	-0.07**	-0.05***	-0.04**	-0.12***	
	(0.08)	(0.03)	(0.02)	(0.02)	(0.03)	
2	-0.37***	-0.09**	-0.03	-0.02	-0.12***	
	(0.09)	(0.03)	(0.02)	(0.03)	(0.04)	
3	-0.47***	-0.03	-0.01	0.02	-0.14***	
	(0.15)	(0.03)	(0.02)	(0.02)	(0.04)	
Observations	9853	9871	10915	9701	9869	

Notes: eTable 6 shows the full set of coefficients estimated from the event-study specification. The unit of analysis is HHA-years. "All Transactions" indicate all types of HHA CHOW transactions, including both changes within the for-profit status and changes from nonprofit or public to for-profit status, compared with all matched control HHAs (for-profit, nonprofit, and public) that never had CHOW during the study period. We limited the analyses to HHA-years with non-missing outcomes for each outcome; the corresponding observations for each outcome are reported. Pre-average is the dynamic aggregation of average treatment effects on the treated across 4 years before the transaction. Post-average is the dynamic aggregation of average treatment effects on the treated across 3 years following the transaction. ***p<0.001, ** p<0.05, * p<0.1.

© 2024 Zhang Z et al. JAMA Health Forum.

Abbreviations: HHA, home health agency; CHOW, change of ownership; FTE, full-time equivalent; PT, physical therapy; OT, occupational therapy; LPN, licensed practical nurse; LVN, licensed vocational nurse.

© 2024 Zhang Z et al. JAMA Health Forum.

eTable 7. Quality of Care Outcomes for HHAs with Change of Ownership Transactions and Matched Controls (FP-FP Transactions)

Outcome	Composite Star Ratings	Timely Initiation of care	Improvement in Ambulation	Improvement in Bed Transferring	Improvement in Bathing
	Coefficient (SE)	Coefficient (SE)	Coefficient (SE)	Coefficient (SE)	Coefficient (SE)
Pre Average	0.01	-0.28	0.44	0.42	0.61
	(0.05)	(0.29)	(0.49)	(0.60)	(0.55)
Post Average	0.11	-0.16	1.71	0.88	1.61*
	(0.08)	(0.37)	(1.06)	(1.40)	(0.84)
Time Period					
-5	0.04	-0.18	0.17	0.20	0.49
	(0.05)	(0.35)	(0.60)	(0.69)	(0.60)
-4	-0.00	-0.57*	0.68	0.52	0.75
	(0.06)	(0.34)	(0.50)	(0.56)	(0.50)
-3	-0.03	-0.30	0.44	0.26	0.42
	(0.06)	(0.25)	(0.38)	(0.44)	(0.45)
-2	0.04	-0.04	0.91	0.60	1.08*
	(0.07)	(0.17)	(0.64)	(0.88)	(0.62)
0	-0.02	0.00	0.14	-0.06	0.46
	(0.06)	(0.24)	(0.34)	(0.36)	(0.44)
1	0.03	0.05	0.40	0.27	0.87*
	(0.04)	(0.24)	(0.43)	(0.42)	(0.48)
4	-0.03	-1.14**	0.89	0.21	0.67
	(0.08)	(0.55)	(0.93)	(1.14)	(0.82)
8	0.19*	-0.06	2.32	1.36	2.63**
	(0.11)	(0.59)	(1.48)	(2.07)	(1.26)
12	0.51***	1.19	5.05**	3.57	3.00*
	(0.17)	(0.95)	(2.32)	(2.52)	(1.78)
Observations	30451	30451	30451	30451	30451
Outcome	Improvement in Dyspnea	Improvement in Management of Oral Medication	60-day Acute Care Hospitalization	60-day Outpatient ED Use	
	Coefficient (SE)	Coefficient (SE)	Coefficient (SE)	Coefficient (SE)	
Pre Average	0.70	0.78	0.07	0.09	
	(0.55)	(0.56)	(0.17)	(0.14)	
Post Average	1.91	2.36*	0.20	-0.05	
	(1.36)	(1.28)	(0.20)	(0.20)	
Time period					
-5	0.64	0.30	0.06	0.15	
	(0.69)	(0.75)	(0.23)	(0.19)	
-4	0.42	0.70	0.19	0.01	
	(0.59)	(0.63)	(0.20)	(0.17)	
-3	0.59	0.72	0.06	-0.05	
	(0.46)	(0.56)	(0.16)	(0.15)	
-2	0.69	1.24	-0.11	0.21	
	(0.76)	(0.79)	(0.16)	(0.16)	
0	0.07	-0.01	-0.30	0.17	
	(0.49)	(0.64)	(0.21)	(0.13)	
1	0.53	0.75	-0.08	-0.09	
	(0.46)	(0.56)	(0.21)	(0.17)	
4	0.63	1.19	0.51*	-0.19	
	(1.25)	(1.11)	(0.27)	(0.23)	
8	3.18*	3.54**	0.46	0.03	
	(1.92)	(1.79)	(0.33)	(0.28)	
12	4.11	6.64**	0.37	-0.23	
	(2.93)	(2.72)	(0.40)	(0.51)	
Observations	30451	30451	30451	30451	

Notes: eTable 7 shows the full set of coefficients estimated from the event-study specification (selected time periods were reported for simplicity; full results available upon request). The unit of analysis is HHA-quarters. "FP-FP Transactions" indicate an ownership transaction within the for-profit status, compared with matched for-profit HHAs that never had CHOW during the study period. Pre-average is the dynamic aggregation of average treatment effects

© 2024 Zhang Z et al. JAMA Health Forum.

on the treated across 8 quarters before the transaction. Post-average is the dynamic aggregation of average treatment effects on the treated across 12 quarters following the transaction. ***p<0.001, ** p<0.05, * p<0.1.
Abbreviations: FP, for-profit; NFP, non-profit or public; HHA, home health agency; CHOW, change of ownership; ED, emergency department.

© 2024 Zhang Z et al. JAMA Health Forum.

eTable 8. Patient Volumes, Per-capita Payment, and Staffing Outcomes for HHAs with Change of Ownership Transactions and Matched Controls (FP-FP Transactions)

Outcome	Medicare Beneficiaries	Per-capita Payment	Registered Nurse FTE	LPN/LVN FTE	Physical Therapy FTE	Occupational Therapy FTE
	Coefficient (SE)	Coefficient (SE)	Coefficient (SE)	Coefficient (SE)	Coefficient (SE)	Coefficient (SE)
Pre Average	0.02	0.01	0.02	0.02	0.07	0.04
	(0.05)	(0.01)	(0.04)	(0.16)	(0.09)	(0.07)
Post Average	0.04	0.04	-0.14**	-0.46***	-0.09	0.09
	(0.05)	(0.03)	(0.06)	(0.10)	(0.10)	(0.10)
Time Period						
-4	0.03	0.02	0.02	-0.35	-0.01	0.03
	(0.08)	(0.01)	(0.07)	(0.23)	(0.23)	(0.17)
-3	0.02	0.01	0.06	0.19	0.20***	0.09*
	(0.05)	(0.02)	(0.04)	(0.29)	(0.06)	(0.05)
-2	0.01	0.01	-0.02	0.21***	0.03	-0.01
	(0.02)	(0.01)	(0.03)	(0.04)	(0.04)	(0.05)
0	-0.05*	0.01	-0.04	-0.08	-0.09*	0.07
	(0.03)	(0.01)	(0.03)	(0.07)	(0.05)	(0.06)
1	0.04	0.06**	-0.09	-0.33***	-0.07	0.12
	(0.05)	(0.03)	(0.06)	(0.10)	(0.11)	(0.11)
2	0.10*	0.08**	-0.12	-0.52***	-0.15	0.11
	(0.06)	(0.03)	(0.08)	(0.13)	(0.14)	(0.14)
3	0.05	0.02	-0.29*	-0.91***	-0.06	0.07
	(0.11)	(0.06)	(0.15)	(0.22)	(0.19)	(0.23)
Observations[b]	8846	8846	8804	7061	5189	3844
Outcome	Home Health Aide FTE	Minutes per Skilled Nursing Visit	Minutes per Physical Therapy Visit	Minutes per Occupational Therapy Visit	Minutes Per Home Health Aide Visit	
	Coefficient (SE)	Coefficient (SE)	Coefficient (SE)	Coefficient (SE)	Coefficient (SE)	
Pre Average	-0.02	0.00	0.01	0.01	-0.00	
	(0.07)	(0.02)	(0.01)	(0.02)	(0.02)	
Post Average	-0.26***	-0.08***	-0.04***	-0.02	-0.10***	
	(0.08)	(0.02)	(0.01)	(0.02)	(0.02)	
Time period						
-4	-0.20*	0.02	0.01	0.01	0.01	
	(0.11)	(0.03)	(0.03)	(0.04)	(0.03)	
-3	0.12	0.01	0.02	0.03*	0.00	
	(0.13)	(0.02)	(0.01)	(0.02)	(0.02)	
-2	0.04	-0.02	0.00	0.01	-0.01	
	(0.05)	(0.02)	(0.01)	(0.01)	(0.02)	
0	-0.04	-0.04**	-0.01	-0.00	-0.02	
	(0.05)	(0.02)	(0.01)	(0.01)	(0.02)	
1	-0.13	-0.09***	-0.06***	-0.05***	-0.10***	
	(0.09)	(0.03)	(0.02)	(0.02)	(0.03)	
2	-0.37***	-0.14***	-0.05***	-0.03	-0.12***	
	(0.11)	(0.04)	(0.02)	(0.02)	(0.04)	
3	-0.50***	-0.05*	-0.02	0.01	-0.14***	
	(0.16)	(0.03)	(0.02)	(0.02)	(0.04)	
Observations	7923	8086	8834	7955	7882	

Notes: eTable 8 shows the full set of coefficients estimated from the event-study specification. The unit of analysis is HHA-years. “FP-FP Transactions” indicate an ownership transaction within the for-profit status, compared with matched for-profit HHAs that never had CHOW during the study period. We limited the analyses to HHA-years with non-missing outcomes for each outcome; the corresponding observations for each outcome are reported. Pre-average is the dynamic aggregation of average treatment effects on the treated across 4 years before the transaction. Post-average is the dynamic aggregation of average treatment effects on the treated across 3 years following the transaction. ***p<0.001, ** p<0.05, * p<0.1.

© 2024 Zhang Z et al. JAMA Health Forum.

Abbreviations: FP, for-profit; NFP, non-profit or public; HHA, home health agency; CHOW, change of ownership; FTE, full-time equivalent; PT, physical therapy; OT, occupational therapy; LPN, licensed practical nurse; LVN, licensed vocational nurse.

© 2024 Zhang Z et al. JAMA Health Forum.

eTable 9. Quality of Care Outcomes for HHAs with Change of Ownership Transactions and Matched Controls (NFP-FP Transactions)

Outcome	Composite Star Ratings	Timely Initiation of care	Improvement in Ambulation	Improvement in Bed Transferring	Improvement in Bathing
	Coefficient (SE)	Coefficient (SE)	Coefficient (SE)	Coefficient (SE)	Coefficient (SE)
Pre Average	0.09	-0.76*	0.54	1.38	0.19
	(0.07)	(0.43)	(0.61)	(0.99)	(0.42)
Post Average	0.39***	1.91***	-0.77	-0.06	0.17
	(0.10)	(0.54)	(1.41)	(1.60)	(1.48)
Time Period					
-5	0.09	-0.89*	0.98	2.21*	0.82*
	(0.07)	(0.49)	(0.71)	(1.29)	(0.45)
-4	0.05	-1.58***	0.96*	2.29**	0.57
	(0.07)	(0.47)	(0.52)	(0.99)	(0.38)
-3	0.06	-0.80	0.44	1.49	-0.42
	(0.08)	(0.63)	(0.58)	(0.92)	(0.54)
-2	0.10	-0.65**	-0.00	0.97*	-0.06
	(0.07)	(0.26)	(0.33)	(0.52)	(0.26)
0	0.10	0.19	-0.38	-0.23	-0.38
	(0.08)	(0.45)	(0.36)	(0.53)	(0.43)
1	0.15*	0.84	-0.77**	-0.13	-0.46
	(0.08)	(0.65)	(0.38)	(0.48)	(0.39)
4	0.40	-0.39	0.75	2.52	1.34
	(0.25)	(0.64)	(2.08)	(2.17)	(2.46)
8	0.69***	3.78***	2.58	1.87	2.18
	(0.21)	(1.26)	(2.14)	(1.94)	(1.80)
12	0.53**	3.48***	-5.46	-5.39	-2.19
	(0.23)	(0.94)	(4.67)	(4.70)	(5.06)
Observations	7392	7392	7392	7392	7392
Outcome	Improvement in Dyspnea	Improvement in Management of Oral Medication	60-day Acute Care Hospitalization	60-day Outpatient ED Use	
	Coefficient (SE)	Coefficient (SE)	Coefficient (SE)	Coefficient (SE)	
Pre Average	0.54	0.62	-0.03	-0.02	
	(0.53)	(0.58)	(0.25)	(0.22)	
Post Average	0.96	-0.09	-0.15	-0.26	
	(1.47)	(1.65)	(0.26)	(0.34)	
Time period					
-5	1.03	1.44**	-0.13	0.10	
	(0.71)	(0.57)	(0.36)	(0.27)	
-4	0.70	0.76	-0.08	-0.17	
	(0.52)	(0.55)	(0.21)	(0.25)	
-3	-0.62	0.43	0.03	-0.02	
	(0.55)	(0.57)	(0.18)	(0.24)	
-2	-0.04	0.28	0.19	-0.09	
	(0.28)	(0.38)	(0.18)	(0.17)	
0	0.14	-0.72*	-0.09	-0.04	
	(0.39)	(0.39)	(0.15)	(0.16)	
1	0.21	-1.08*	0.01	0.15	
	(0.40)	(0.57)	(0.20)	(0.21)	
4	1.87	1.51	-0.41	-0.24	
	(2.03)	(3.06)	(0.40)	(0.42)	
8	3.24	2.45	-0.31	-0.12	
	(2.36)	(1.93)	(0.39)	(0.45)	
12	-0.24	-3.91	0.08	-1.21	
	(4.62)	(5.11)	(0.57)	(1.14)	
Observations	7392	7392	7392	7392	

Notes: eTable 9 shows the full set of coefficients estimated from the event-study specification (selected time periods were reported for simplicity; full results available upon request). The unit of analysis is HHA-quarters. "NFP-FP Transactions" indicate an ownership transaction from nonprofit or public to for-profit status, compared with matched nonprofit or public HHAs that never had CHOW during the study period. Pre-average is the dynamic aggregation of

© 2024 Zhang Z et al. JAMA Health Forum.

average treatment effects on the treated across 8 quarters before the transaction. Post-average is the dynamic aggregation of average treatment effects on the treated across 12 quarters following the transaction. ***p<0.001, ** p<0.05, * p<0.1.
Abbreviations: FP, for-profit; NFP, non-profit or public; HHA, home health agency; CHOW, change of ownership; ED, emergency department.

© 2024 Zhang Z et al. JAMA Health Forum.

eTable 10. Patient Volumes, Per-capita Payment, and Staffing Outcomes for HHAs with Change of Ownership Transactions and Matched Controls (NFP-FP Transactions)

Outcome	Medicare Beneficiaries	Per-capita Payment	Registered Nurse FTE	LPN/LVN FTE	Physical Therapy FTE	Occupational Therapy FTE
	Coefficient (SE)	Coefficient (SE)	Coefficient (SE)	Coefficient (SE)	Coefficient (SE)	Coefficient (SE)
Pre Average	0.05	-0.01	0.03	0.14	-0.17	0.03
	(0.06)	(0.02)	(0.06)	(0.25)	(0.13)	(0.09)
Post Average	-0.11	0.06	-0.15	0.45	-0.06	0.27
	(0.09)	(0.04)	(0.18)	(0.36)	(0.15)	(0.20)
Time Period						
-4	-0.02	-0.02	0.03	0.23	-0.34	0.16
	(0.07)	(0.03)	(0.11)	(0.43)	(0.27)	(0.17)
-3	0.04	0.01	-0.02	0.04	-0.16	0.03
	(0.12)	(0.02)	(0.07)	(0.28)	(0.17)	(0.10)
-2	0.14**	-0.01	0.07	0.13	-0.02	-0.11
	(0.06)	(0.01)	(0.04)	(0.26)	(0.06)	(0.08)
0	-0.36***	-0.00	-0.27*	0.56	-0.07*	0.23
	(0.09)	(0.02)	(0.14)	(0.37)	(0.04)	(0.21)
1	-0.19*	0.09**	-0.51***	0.27	-0.37**	0.05
	(0.11)	(0.04)	(0.17)	(0.32)	(0.16)	(0.12)
2	-0.07	0.08	-0.24	0.51	-0.13	0.24*
	(0.12)	(0.05)	(0.18)	(0.58)	(0.12)	(0.14)
3	0.18	0.08	0.42	0.46	0.35	0.55
	(0.22)	(0.10)	(0.47)	(0.87)	(0.45)	(0.55)
Observations[b]	2088	2088	2088	1327	1556	1242
Outcome	Home Health Aide FTE	Minutes per Skilled Nursing Visit	Minutes per Physical Therapy Visit	Minutes per Occupational Therapy Visit	Minutes Per Home Health Aide Visit	
	Coefficient (SE)	Coefficient (SE)	Coefficient (SE)	Coefficient (SE)	Coefficient (SE)	
Pre Average	0.08	-0.00	0.00	0.01	0.00	
	(0.11)	(0.02)	(0.02)	(0.01)	(0.02)	
Post Average	-0.22	0.02	-0.00	0.02	-0.14**	
	(0.14)	(0.04)	(0.03)	(0.04)	(0.06)	
Time period						
-4	0.22	0.00	0.02	0.02	0.05***	
	(0.21)	(0.02)	(0.04)	(0.02)	(0.02)	
-3	0.03	0.01	-0.00	0.02	-0.02	
	(0.10)	(0.04)	(0.02)	(0.02)	(0.03)	
-2	-0.02	-0.02	-0.02	-0.02	-0.03*	
	(0.05)	(0.03)	(0.01)	(0.02)	(0.02)	
0	-0.14	0.00	-0.02	-0.01	-0.11**	
	(0.10)	(0.04)	(0.02)	(0.02)	(0.05)	
1	-0.13	0.02	-0.02	-0.02	-0.20**	
	(0.16)	(0.06)	(0.05)	(0.05)	(0.08)	
2	-0.37***	0.07	0.02	0.02	-0.12	
	(0.14)	(0.07)	(0.06)	(0.08)	(0.09)	
3	-0.24	-0.03	0.01	0.08	-0.12	
	(0.37)	(0.03)	(0.02)	(0.07)	(0.13)	
Observations	1930	1785	2081	1746	1987	

Notes: eTable 10 shows the full set of coefficients estimated from the event-study specification. The unit of analysis is HHA-years. "NFP-FP Transactions" indicate an ownership transaction from nonprofit or public to for-profit status, compared with matched nonprofit or public HHAs that never had CHOW during the study period. We limited the analyses to HHA-years with non-missing outcomes for each outcome; the corresponding observations for each outcome are reported. Pre-average is the dynamic aggregation of average treatment effects on the treated across 4 years before the transaction. Post-average is the dynamic aggregation of average treatment effects on the treated across 3 years following the transaction. ***p<0.001, ** p<0.05, * p<0.1.

© 2024 Zhang Z et al. JAMA Health Forum.

Abbreviations: FP, for-profit; NFP, non-profit or public; HHA, home health agency; CHOW, change of ownership; FTE, full-time equivalent; PT, physical therapy; OT, occupational therapy; LPN, licensed practical nurse; LVN, licensed vocational nurse.

© 2024 Zhang Z et al. JAMA Health Forum.

eTable 11. Baseline Characteristics of HHAs with Change of Ownership Transactions and Unmatched Controls[a]

	Without CHOW (Unmatched Control)[b,c]	With CHOW[c]	P-Value[d]
No. of HHAs	7163	294	
Pre-transaction ownership, No. (%)			
For Profit	5789 (81)	235 (80)	0.71
Nonprofit	1030 (14)	41 (14)	0.84
Government	340 (5)	18 (6)	0.28
Include any branch, No. (%)	802 (11)	69 (23)	0.00
Operate hospice, No. (%)	403 (6)	22 (7)	0.18
Region, No. (%)			
Northeast	559 (8)	23 (8)	0.99
Midwest	2035 (28)	46 (16)	0.00
South	2955 (41)	189 (64)	0.00
West	1581 (22)	36 (12)	0.00
HHA Quality of Care, mean (SD)			
Composite Star Ratings	3.28 (0.88)	3.35 (0.79)	0.12
Timely Initiation of Care	92.57 (7.97)	92.33 (6.96)	0.55
Improvement in Ambulation	68.2 (13.83)	67.43 (10.83)	0.18
Improvement in Bed Transferring	64.33 (15.8)	64.23 (12.88)	0.89
Improvement in Bathing	71.06 (14.69)	71.32 (11.78)	0.68
Improvement in Dyspnea	67.41 (18.69)	68.21 (14.36)	0.3
Improvement in Management of Oral Medications	56.91 (16.31)	57.33 (12.6)	0.53
Rate of 60-Day Acute Care Hospitalization	15.37 (4.15)	16.12 (3.7)	0.00
Rate of 60-Day ED Visits	12.21 (4.27)	13.09 (3.98)	0.00
Size and Capacity, mean (SD)			
Number of Medicare Beneficiaries	346 (556)	538 (998)	0.01
Per-Capita Medicare Payment	5662 (2070)	5115 (1654)	0.00
Patient Characteristics, mean (SD)			
Dual	37.03 (24.52)	23.36 (13.49)	0.00
Rural	27.32 (36.63)	42.54 (38.41)	0.00
Racial and Ethnic Minoritized Group	31.33 (26.37)	20.87 (18.39)	0.00
Average HCC Score	2.37 (0.47)	2.3 (0.4)	0.00
Staffing, mean (SD)			
Registered Nurse FTE	8.73 (74.49)	13.58 (39.14)	0.05
LPN/LVN FTE	2.78 (10.86)	3.58 (9.96)	0.16
PT FTE	2.68 (7.4)	4.08 (9.3)	0.01
OT FTE	0.93 (3.2)	1.14 (2.24)	0.17
Home Health Aide FTE	3.84 (18.62)	6.05 (25.51)	0.19
Minutes of Skilled Nursing Care Per Visit	47.36 (10.17)	46.98 (8.54)	0.45
Minutes of PT Per Visit	47.37 (8.15)	46.63 (7.2)	0.07
Minutes of OT Per Visit	47.97 (8.48)	47.15 (8.09)	0.08
Minutes of Home Health Aide Per Visit	56.6 (15.91)	56.13 (16.93)	0.67

Notes: [a]The unit of analysis for quality-of-care outcomes is HHA-quarters. The unit of analysis for patient characteristics, volumes and per-capita payments, and staffing outcomes is HHA-years.
[b]Baseline characteristics were derived based on the pre-transaction periods for HHAs with CHOW and all time periods for control HHAs during 2016-2019.
[d]P-value from a t-test of the difference in means between CHOW status at baseline; standard errors were clustered at the HHA level.
[e]Measures were presented as a percentage of home health episodes. Additional details in eTable 3 in Supplement.
[f]Measures were presented as a percentage of Medicare beneficiaries.
Abbreviations: HHA, home health agency; CHOW, change of ownership; ED, emergency department; HCC, hierarchical condition category; FTE, full-time equivalent; PT, physical therapy; OT, occupational therapy; LPN, licensed practical nurse; LVN, licensed vocational nurse.

© 2024 Zhang Z et al. JAMA Health Forum.

eTable 12. Outcomes for HHAs with Change of Ownership Transactions and Unmatched Controls, by Transaction Types[a]

	All transaction types (n = 294)			For-profit to for-profit HHA (n = 237)			Nonprofit/public to for-profit HHA (n = 57)		
Outcome	Baseline mean (SD)	Estimated Δ (95% CI)[b]	Pretrend (*P* value)[c]	Baseline mean (SD)	Estimated Δ (95% CI)[b]	Pretrend (*P* value)[c]	Baseline mean (SD)	Estimated Δ (95% CI)[b]	Pretrend (*P* value)[c]
**Quality of care, HHA quarters**									
Quality-of-care star rating	3.62 (0.71)	0.18 (0.05, 0.31)	0.16	3.71 (0.74)	0.11 (-0.04, 0.26)	0.38	3.35 (0.53)	0.4 (0.19, 0.61)	0.04
Timely initiation of care, pp	92.70 (5.60)	0.38 (-0.25, 1.01)	0.01	93.67 (5.09)	-0.21 (-0.94, 0.51)	0.11	89.77 (6.06)	2.24 (1.19, 3.29)	0.00
Improvement in ambulation, pp	70.92 (8.92)	1.31 (-0.44, 3.05)	0.18	71.91 (9.48)	1.78 (-0.28, 3.84)	0.24	67.89 (5.99)	-0.51 (-3.26, 2.24)	0.34
Improvement in bed transferring, pp	67.94 (10.08)	0.67 (-1.58, 2.92)	0.26	68.24 (10.75)	0.87 (-1.86, 3.6)	0.67	67.05 (7.65)	-0.01 (-3.09, 3.08)	0.07
Improvement in bathing, pp	74.89 (8.99)	1.38 (-0.03, 2.78)	0.13	75.65 (9.63)	1.69 (0.06, 3.31)	0.26	72.60 (6.17)	0.36 (-2.53, 3.25)	0.12
Improvement in dyspnea, pp	73.47 (10.74)	1.78 (-0.39, 3.95)	0.83	73.47 (11.70)	1.82 (-0.81, 4.45)	0.78	73.46 (7.10)	1.09 (-1.7, 3.87)	0.04
Improvement in management of oral medications, pp	62.06 (10.48)	2.01 (-0.09, 4.1)	0.50	62.64 (11.34)	2.42 (-0.07, 4.9)	0.38	60.28 (7.02)	0.08 (-3.16, 3.32)	0.00
Rate of 60-d acute care hospitalization, pp	16.75 (2.57)	0.18 (-0.15, 0.5)	0.77	16.56 (2.53)	0.26 (-0.12, 0.65)	0.36	17.31 (2.60)	-0.11 (-0.62, 0.41)	0.78
Rate of 60-d outpatient ED use, pp	12.43 (2.64)	-0.09 (-0.43, 0.25)	0.30	12.61 (2.62)	-0.02 (-0.41, 0.37)	0.31	11.92 (2.64)	-0.34 (-0.99, 0.32)	0.57
**Volume and per-capita payment, HHA years[d]**									
No. of Medicare beneficiaries	7.07 (1.30)	0.01 (-0.09, 0.1)	0.51	6.97 (1.34)	0.04 (-0.06, 0.13)	0.99	7.35 (1.13)	-0.04 (-0.22, 0.14)	0.05
Medicare per-capita payment	8.42 (0.26)	0.05 (0, 0.09)	0.68	8.48 (0.25)	0.04 (-0.02, 0.1)	0.64	8.23 (0.18)	0.07 (-0.01, 0.15)	0.07
**Staffing[d]**									
Registered nurse FTE	3.07 (1.40)	-0.14 (-0.28, 0)	0.81	2.97 (1.46)	-0.11 (-0.24, 0.02)	0.37	3.36 (1.16)	-0.11 (-0.48, 0.25)	0.10
LPN/LVN FTE	1.44 (1.38)	-0.33 (-0.55, -0.1)	0.03	1.51 (1.47)	-0.46 (-0.65, -0.26)	0.00	1.21 (0.97)	0.4 (-0.3, 1.11)	0.80
Physical therapist FTE	2.19 (1.30)	-0.09 (-0.26, 0.07)	0.15	2.16 (1.36)	-0.1 (-0.3, 0.09)	0.00	2.29 (1.05)	0.01 (-0.28, 0.3)	0.10
Occupational therapist FTE	1.13 (1.20)	0.11 (-0.07, 0.3)	0.05	1.14 (1.25)	0.09 (-0.11, 0.29)	0.03	1.11 (1.01)	0.22 (-0.16, 0.61)	0.32
Home health aide FTE	1.44 (1.46)	-0.23 (-0.36, -0.11)	0.25	1.47 (1.60)	-0.23 (-0.38, -0.09)	0.10	1.35 (0.97)	-0.2 (-0.47, 0.07)	0.58
Skilled nursing care, min/visit	3.82 (0.19)	-0.05 (-0.09, -0.01)	0.59	3.80 (0.17)	-0.07 (-0.12, -0.03)	0.28	3.87 (0.24)	0.01 (-0.06, 0.08)	0.38
Physical therapy, min/visit	3.82 (0.17)	-0.02 (-0.05, 0)	0.83	3.81	-0.03 (-0.06, -0.01)	0.66	3.85 (0.22)	0 (-0.06, 0.07)	0.12
Occupational therapy, min/visit	3.82 (0.18)	-0.01 (-0.04, 0.02)	0.15	3.81	-0.01 (-0.04, 0.02)	0.14	3.86 (0.23)	0.02 (-0.06, 0.11)	0.36
Home health aide care, min/visit	3.93 (0.30)	-0.1 (-0.14, -0.06)	0.38	3.88	-0.09 (-0.14, -0.04)	0.92	4.06 (0.30)	-0.12 (-0.23, -0.01)	0.00

© 2024 Zhang Z et al. JAMA Health Forum.

Abbreviations: ED, emergency department; FTE, full-time equivalent; HHA, home health agency; LPN, licensed practical nurse; LVN, licensed vocational nurse; pp, percentage point.

[a]Each observation (HHA quarters or HHA years) was weighted by the total number of unique patients served by Medicare in the first observation year of the study period. Pretreatment quarters differed for HHAs that changed ownership at different periods.

[b]Estimates represent the dynamic aggregation of average treatment effects on the treated across 12 quarters posttransaction.

[c]Pretrend tests report the *P* value from a joint F-test of the estimated coefficients pretransaction. Significant test results ($P < 0.05$) suggest evidence of pretrend.

[d]Logarithmically transformed. For HHA year analyses, the observation was further limited with nonmissing outcomes for each regression.

© 2024 Zhang Z et al. JAMA Health Forum.

eTable 13. Outcomes for HHAs with Change of Ownership Transactions and Matched Controls, by Transaction Types (without Detrending)[a]

	All transaction types (n = 294)			For-profit to for-profit HHA (n = 237)			Nonprofit/public to for-profit HHA (n = 57)		
Outcome	Baseline mean (SD)	Estimated Δ (95% CI)[b]	Pretrend (*P* value)[c]	Baseline mean (SD)	Estimated Δ (95% CI)[b]	Pretrend (*P* value)[c]	Baseline mean (SD)	Estimated Δ (95% CI)[b]	Pretrend (*P* value)[c]
**Quality of care, HHA quarters**									
Quality-of-care star rating	3.62 (0.71)	0.08 (-0.05, 0.21)	0.04	3.71 (0.74)	0.02 (-0.13, 0.18)	0.29	3.35 (0.53)	0.24 (0.04, 0.45)	0.08
Timely initiation of care, pp	92.70 (5.60)	0.47 (-0.16, 1.1)	0.01	93.67 (5.09)	0.13 (-0.6, 0.86)	0.11	89.77 (6.06)	1.6 (0.53, 2.66)	0.01
Improvement in ambulation, pp	70.92 (8.92)	0.64 (-1.12, 2.39)	0.10	71.91 (9.48)	0.8 (-1.28, 2.89)	0.17	67.89 (5.99)	-0.4 (-3.15, 2.36)	0.26
Improvement in bed transferring, pp	67.94 (10.08)	-0.49 (-2.75, 1.77)	0.07	68.24 (10.75)	-0.8 (-3.56, 1.95)	0.31	67.05 (7.65)	0.04 (-3.1, 3.17)	0.04
Improvement in bathing, pp	74.89 (8.99)	0.06 (-1.36, 1.47)	0.01	75.65 (9.63)	0.01 (-1.64, 1.65)	0.05	72.60 (6.17)	-0.02 (-2.92, 2.88)	0.06
Improvement in dyspnea, pp	73.47 (10.74)	-0.15 (-2.33, 2.02)	0.02	73.47 (11.70)	-0.14 (-2.8, 2.52)	0.06	73.46 (7.10)	-0.69 (-3.57, 2.18)	0.00
Improvement in management of oral medications, pp	62.06 (10.48)	1.21 (-0.89, 3.32)	0.31	62.64 (11.34)	1.61 (-0.89, 4.12)	0.39	60.28 (7.02)	-0.6 (-3.84, 2.63)	0.00
Rate of 60-d acute care hospitalization, pp	16.75 (2.57)	-0.2 (-0.53, 0.12)	0.47	16.56 (2.53)	-0.16 (-0.55, 0.23)	0.16	17.31 (2.60)	-0.33 (-0.85, 0.18)	0.79
Rate of 60-d outpatient ED use, pp	12.43 (2.64)	0.11 (-0.23, 0.45)	0.39	12.61 (2.62)	0.14 (-0.25, 0.53)	0.38	11.92 (2.64)	-0.05 (-0.71, 0.61)	0.57
**Volume and per-capita payment, HHA years[d]**									
No. of Medicare beneficiaries	7.07 (1.30)	0.05 (-0.04, 0.14)	0.53	6.97 (1.34)	0.04 (-0.06, 0.14)	0.98	7.35 (1.13)	0.14 (-0.04, 0.32)	0.00
Medicare per-capita payment	8.42 (0.26)	0.08 (0.03, 0.13)	0.09	8.48 (0.25)	0.08 (0.02, 0.14)	0.34	8.23 (0.18)	0.09 (0.02, 0.17)	0.01
**Staffing[d]**									
Registered nurse FTE	3.07 (1.40)	-0.12 (-0.26, 0.02)	0.85	2.97 (1.46)	-0.16 (-0.28, -0.03)	0.19	3.36 (1.16)	0.09 (-0.27, 0.45)	0.05
LPN/LVN FTE	1.44 (1.38)	0.11 (-0.11, 0.34)	0.01	1.51 (1.47)	0.02 (-0.18, 0.21)	0.00	1.21 (0.97)	0.71 (0.01, 1.42)	0.85
Physical therapist FTE	2.19 (1.30)	-0.04 (-0.21, 0.12)	0.35	2.16 (1.36)	-0.07 (-0.26, 0.13)	0.01	2.29 (1.05)	0.08 (-0.21, 0.38)	0.00
Occupational therapist FTE	1.13 (1.20)	0.06 (-0.12, 0.25)	0.04	1.14 (1.25)	0.02 (-0.19, 0.22)	0.01	1.11 (1.01)	0.29 (-0.09, 0.67)	0.43
Home health aide FTE	1.44 (1.46)	-0.16 (-0.29, -0.03)	0.13	1.47 (1.60)	-0.17 (-0.32, -0.02)	0.05	1.35 (0.97)	-0.11 (-0.38, 0.16)	0.46
Skilled nursing care, min/visit	3.82 (0.19)	-0.05 (-0.09, -0.01)	0.56	3.80 (0.17)	-0.06 (-0.1, -0.01)	0.21	3.87 (0.24)	-0.04 (-0.11, 0.04)	0.00
Physical therapy, min/visit	3.82 (0.17)	-0.02 (-0.05, 0)	0.70	3.81	-0.03 (-0.05, 0)	0.65	3.85 (0.22)	-0.02 (-0.08, 0.05)	0.31
Occupational therapy, min/visit	3.82 (0.18)	-0.02 (-0.05, 0.01)	0.04	3.81	-0.02 (-0.05, 0.01)	0.03	3.86 (0.23)	0 (-0.09, 0.08)	0.02

© 2024 Zhang Z et al. JAMA Health Forum.

Home health aide care, min/visit	3.93 (0.30)	-0.1 (-0.15, -0.06)	0.50	3.88	-0.09 (-0.14, -0.04)	0.92	4.06 (0.30)	-0.14 (-0.25, -0.02)	0.00

Abbreviations: ED, emergency department; FTE, full-time equivalent; HHA, home health agency; LPN, licensed practical nurse; LVN, licensed vocational nurse; pp, percentage point.

[a]Each observation (HHA quarters or HHA years) was weighted by the total number of unique patients served by Medicare in the first observation year of the study period. Pretreatment quarters differed for HHAs that changed ownership at different periods.

[b]Estimates represent the dynamic aggregation of average treatment effects on the treated across 12 quarters posttransaction.

[c]Pretrend tests report the *P* value from a joint F-test of the estimated coefficients pretransaction. Significant test results ($P < 0.05$) suggest evidence of pretrend.

[d]Logarithmically transformed. For HHA year analyses, the observation was further limited with nonmissing outcomes for each regression.

© 2024 Zhang Z et al. JAMA Health Forum.

eReferences

1. Centers for Medicare & Medicaid Services. Quality of Patient Care Star Ratings Methodology. Home Health Star Ratings. April 2020. Accessed April 8, 2024. https://www.cms.gov/files/document/quality-patient-care-star-ratings-methodologyapril-2020.pdf

2. Provider of Services File - Hospital & Non-Hospital Facilities - Centers for Medicare & Medicaid Services Data. Accessed April 8, 2024. https://data.cms.gov/provider-characteristics/hospitals-and-other-facilities/provider-of-services-file-hospital-non-hospital-facilities

3. Centers for Medicare & Medicaid Services. *Medicare Fee-For-Service Home Health Agency Utilization & Payment Public Use File: A Methodological Overview.*; 2018. Accessed April 8, 2024. https://www.cms.gov/research-statistics-data-and-systems/statistics-trends-and-reports/medicare-provider-charge-data/downloads/hha_methodology.pdf

4. Roth J. Interpreting Event-Studies from Recent Difference-in-Differences Methods. January 22, 2024. Accessed April 8, 2024. https://www.jonathandroth.com/assets/files/HetEventStudies.pdf

5. Kannan S, Bruch JD, Song Z. Changes in Hospital Adverse Events and Patient Outcomes Associated With Private Equity Acquisition. *JAMA*. 2023;330(24):2365. doi:10.1001/jama.2023.23147

6. McConnell KJ, Edelstein S, Hall J, et al. Access, Utilization, and Quality of Behavioral Health Integration in Medicaid Managed Care. *JAMA Health Forum*. 2023;4(12):e234593. doi:10.1001/jamahealthforum.2023.4593

7. Goodman-Bacon A. The Long-Run Effects of Childhood Insurance Coverage: Medicaid Implementation, Adult Health, and Labor Market Outcomes. *Am Econ Rev*. 2021;111(8):2550-2593. doi:10.1257/aer.20171671

8. Agha L, Zeltzer D. *Drug Diffusion Through Peer Networks: The Influence of Industry Payments*. National Bureau of Economic Research; 2019:w26338. doi:10.3386/w26338

9. Centers for Medicare & Medicaid Services. *Medicare Fee-For-Service Provider Enrollment – Home Health Agency (HHA) Change of Ownership: Data Guidance.*; 2023. Accessed December 8, 2023. https://data.cms.gov/sites/default/files/2024-01/HHA_Change%20of%20Ownership_CHOW_Files_2024.01.05.zip

10. Ownership of Skilled Nursing Facilities: An Analysis of Newly-Released Federal Data. ASPE. December 15, 2022. Accessed March 29, 2024. https://aspe.hhs.gov/reports/ownership-skilled-nursing-facilities

© 2024 Zhang Z et al. JAMA Health Forum.
