# Supplementary material for: Change of Ownership and Quality of Home Health Agency Care
Source: JAMA Health Forum. 2024 Nov 1;5(11):e243767. doi: 10.1001/jamahealthforum.2024.3767 (PMC11530943; doi:10.1001/jamahealthforum.2024.3767)
Supplement: Supplement 2. — Data Sharing Statement [file jamahealthforum-e243767-s002.pdf]

Data Sharing Statement

Zhang. Change of Ownership and Quality of Home Health Agency Care. *JAMA Health Forum*. Published November 01, 2024. doi:10.1001/jamahealthforum.2024.3767

Data
Data available: No

Additional Information
Explanation for why data not available: All data used in this study are publicly available. Data on home health agency change of ownership are available via https://data.cms.gov/provider-characteristics/hospitals-and-other-facilities/home-health-agency-all-owners.
